# The Evaluation of the Effects of Two Probiotic Strains on the Oral Ecosystem: A Randomized Clinical Trial

**DOI:** 10.3389/froh.2022.825017

**Published:** 2022-03-30

**Authors:** Catherine M. C. Volgenant, Suzette V. van der Waal, Bernd W. Brandt, Mark J. Buijs, Monique H. van der Veen, N. A. M. Rosema, Bernd L. Fiebich, Thorsten Rose, Tim Schmitter, Max Gajfulin, Wim Crielaard, Egija Zaura

**Affiliations:** ^1^Department of Preventive Dentistry, Academic Centre for Dentistry Amsterdam, Vrije Universiteit Amsterdam and University of Amsterdam, Amsterdam, Netherlands; ^2^Department of Periodontology, Academic Centre for Dentistry Amsterdam, Vrije Universiteit Amsterdam and University of Amsterdam, Amsterdam, Netherlands; ^3^VivaCell Biotechnology GmbH, Denzlingen, Germany; ^4^Symrise AG, Holzminden, Germany; ^5^Spindiag GmbH, Freiburg im Breisgau, Germany

**Keywords:** probiotics, experimental gingivitis, saliva, dental plaque, tongue, *Lactobacillus paracasei*, *Lactobacillus plantarum*

## Abstract

**Introduction:**

In the current study, we evaluated the effectiveness of two well-defined probiotic strains, *Lactobacillus paracasei* LPc-G110 (CCTCC M 2013691) and *Lactobacillus plantarum* GOS42 (DSM 32131), during an experimental gingivitis challenge. The primary objective was to evaluate clinically the effectiveness of lozenges containing one of the two oral probiotic strains, compared with placebo lozenges, on the gingival bleeding (bleeding on marginal probing; BOMP change) after a two-week experimental gingivitis period. The secondary objectives were to assess the effects of the test products on gingival health (Modified Gingival Index; MGI), dental plaque accumulation and fluorescence, and the dynamics of immunological and microbiological aspects after the wash-in phase, followed by a two-week period refraining from oral hygiene and a two-week wash-out phase.

**Methods:**

This single-center challenge intervention study was a triple-blind randomized placebo-controlled clinical trial with three parallel groups. The full study population consisted of 117 healthy 18–55 years old human volunteers. Subjects were instructed to use one lozenge, 3 times daily after each meal, containing either *L. plantarum, L. paracasei*, or lozenges without probiotics (placebo group). After a 2-week wash-in period, the subjects were requested to refrain from any form of oral hygiene for 2 weeks.

**Results:**

There were no differences in the primary outcome (BOMP change) among the groups. However, gingival health (MGI) in individuals from the groups exposed to the test products recovered better from experimental gingivitis than the individuals in the placebo group (*p* = 0.021, one-way ANOVA). The two test products inhibited pro-inflammatory cytokine IL-1ß production, measured in saliva, during the experimental gingivitis period. Both test strains significantly reduced bacterial DNA in tongue samples and *L. paracasei* strain showed stronger microbiome-modulating potential than the *L. plantarum* strain.

**Conclusions:**

The two tested lozenges with the *L. paracasei* or *L. plantarum* strains did show potential for beneficial effects for the oral health of the host during experimental gingivitis to the oral ecosystem.

## Introduction

Probiotics in fermented dairy products, such as yogurt and buttermilk, have been part of the human diet for centuries. In the past several decades, probiotics have been introduced to the market as food or dietary supplements and are often promoted as an important part of nutrition-based health strategies [1].

Probiotics have been used for the prevention and the treatment of a wide range of diseases and they are considered to confer health benefits to the host by beneficially affecting the host's microbial balance and in addition interacting directly with the immune system, modulating its responses [2, 3]. Moreover, many probiotics are generally recognized as safe (GRAS organisms) taking into account that indeed, they have been used in fermentation processes and have therefore been part of our daily nutrition for centuries. The species *Lactobacillus plantarum* and *Lactobacillus paracasei*, used here, are deemed safe for human consumption as they comply with the (QPS) status given by European Food Safety Authority (EFSA).

In 1965, the study of Löe et al. introduced the so-called *experimental gingivitis model* in order to assess oral clinical (resilience) parameters such as (the dynamics of) plaque formation, (the dynamics/onset of) inflammatory patterns, and changes in the oral microbiology [4]. Gingivitis is a reversible, common, and relatively mild form of periodontal disease where the gums of the patient are inflamed. This is reflected in irritation, redness, and swelling of the gums, which bleed more easily when touched. During Löe's experimental gingivitis, healthy volunteers refrained from oral hygiene practices for 3 weeks invoking a general shift from health to a mild inflammatory status. Moreover, poor oral hygiene did not result in long-lasting damage to the gingiva in healthy individuals [4] and the inflammatory status was shown to be reversible following the reintroduction of oral hygiene. Subsequently, several researchers modified the model by reducing the duration to two weeks and demonstrating the validity of the data [5–7].

A recent study used the *experimental gingivitis model* on evaluating an oral probiotic and showed that a probiotic yogurt supplemented with *Bifidobacterium animalis* has a positive effect on plaque accumulation and gingival inflammatory parameters after refraining from oral hygiene practices [8].

In the current study, we evaluated the effectiveness of two well-defined probiotic strains, *L. paracasei* LPc-G110 (CCTCC M 2013691) and *Lactobacillus plantarum* GOS42 (DSM 32131), in strengthening the oral ecosystem. These strains L. selected from a panel of probiotic strains as the first-mentioned was most potent in reducing the proportions of several anaerobic genera in *in vitro* oral biofilms [9, 10] and the latter was most effective in modulation the immune response in a model for gingival immune reactions [11, 12].

The hypothesis of our research was that introduction of the test strains to the oral ecosystem would lead to reduced changes due to abstaining from oral hygiene (experimental gingivitis). The primary objective of this study was to evaluate the clinical effectiveness of two different lozenges containing one of the two oral probiotic strains, respectively, compared with placebo lozenges, on gingival health after a two-week experimental gingivitis period. The secondary objectives were to assess the effects of the test products on dental plaque accumulation and fluorescence, and the dynamics of immunological and microbiological aspects after the wash-in phase, followed by a two-week period refraining from oral hygiene and a two-week wash-out phase.

## Materials and Methods

### Study Settings and Approval

The study was conducted at the dental clinics of the Academic Center for Dentistry Amsterdam (ACTA), the Netherlands. ACTA is the joint faculty of dentistry of the Vrije Universiteit Amsterdam and the University of Amsterdam. The study protocol was approved by the ethics committee of Slotervaartziekenhuis & Reade (METC nr. P1815; CCMO research protocol nr. NL65326.048.18) and the internal scientific research committee of ACTA and is registered in the Netherlands Trial Register under NL6951. The study was performed in accordance with the ethical principles of the 64^th^ WMA Declaration of Helsinki [13] in accordance with the Dutch Medical Research Involving Human Subjects Act (WMO) and in agreement with the Good Clinical Practice [14] guidelines. The GCP compliance and monitoring of the study were conducted by external study monitors from Profess Medical Consultancy BV. All study participants provided written informed consent before participating in the study.

### Study Participants

In order to be eligible to participate in this study, a subject had to meet all of the following criteria: systemically healthy (as assessed by a medical questionnaire); adult (18–55 years); possessing a minimum of 20 natural teeth with at least the first or second molar present in each quadrant; having visited the dentist for a regular check-up within the last year and without any treatment(s) scheduled; and willing and able to give written informed consent and to comply to all study procedures. Subjects were excluded if they did not meet the inclusion criteria or if they were ACTA dental students or ACTA employees. Further exclusion criteria includes the following: if the subjects had participated in another clinical study within the previous 30 days; had allergy/intolerance to the test or placebo products (ingredients), in particular lactose and milk proteins (allergens); were smokers (definition of a non-smoker: <1 cigarette every day for at least 1 year, including e-cigarettes); were drug or alcohol abusers; were pregnant or breastfeeding; had used antibiotics during the last 3 months; required antibiotic prophylaxis prior to dental treatment; were using anti-inflammatory drugs on a regular basis; had any systemic disease or compromised health condition, including diabetes mellitus, bronchitis, tonsillitis or sinusitis, severe oral or pharyngeal infections, and disorders/disease resulting in (self-induced) vomiting; reduced salivary flow due to pathological reasons (e.g., Sjögren syndrome); had adverse medical history or long-term medication; were using prescribed medication (except for contraceptives); were not willing not to consume any other probiotic products during the study. Additionally, specific exclusion criteria based on their oral health were applied: dental pocket probing depth ≥5 mm with bleeding on probing and attachment loss ≥2 mm (Dutch Periodontal Screening Index score 3+/ 4); clearly inflamed gingiva, presented as >40% BOP; overt dental caries; removable partial dentures or nightguard; oral and/or peri-oral piercings; apparent oral lesions (except small aphthous ulcers); orthodontic banding (except lingual retention wires); ongoing or planned elective dental treatment involving endodontic treatment and crown and bridge preparation.

### Study Design

**Figure 1:**
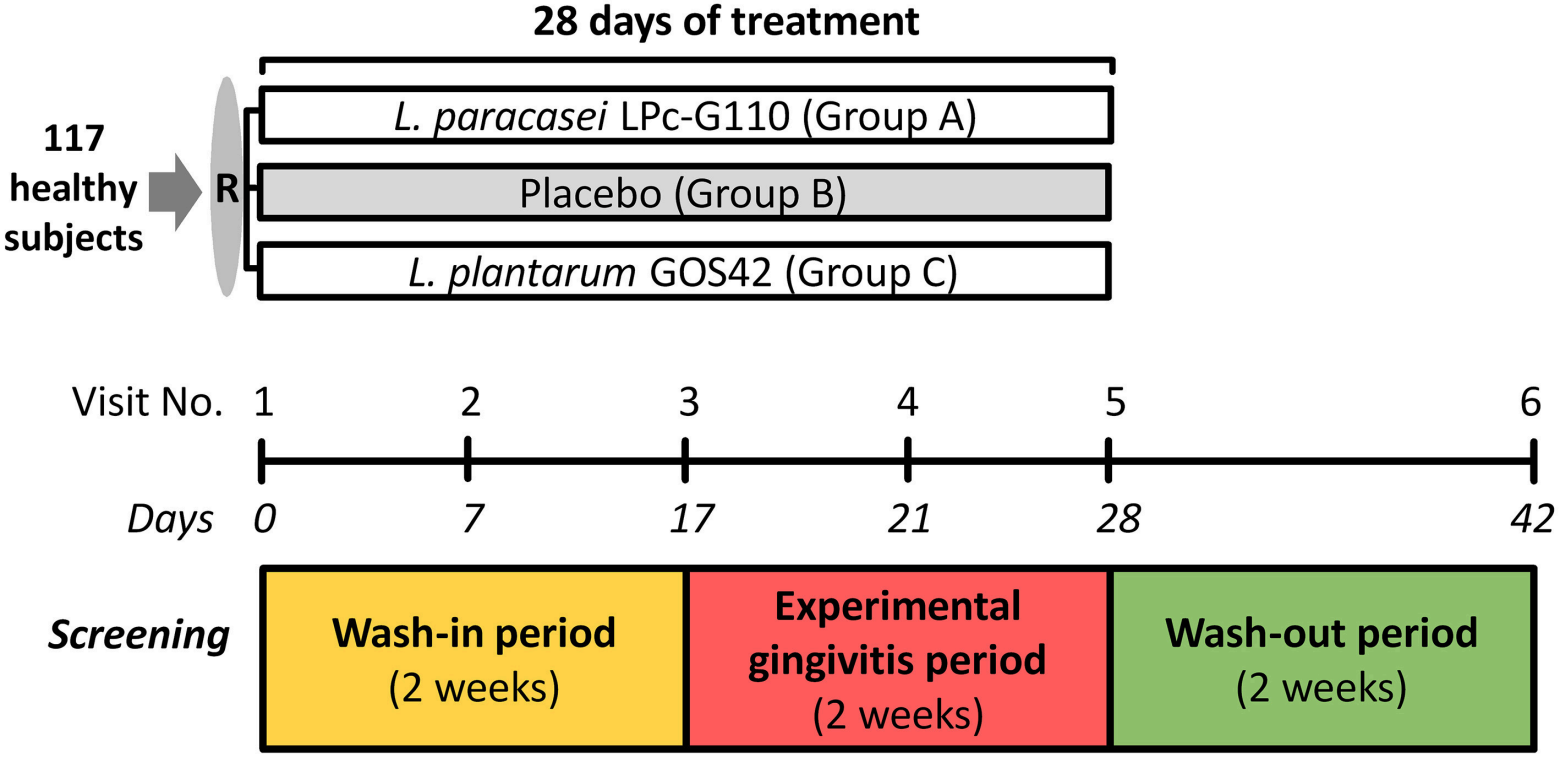
Study outline. R – randomization.

This study was a single-center, challenge intervention, triple-blind, parallel-group (three groups) randomized, and a placebo-controlled clinical trial with 117 healthy subjects (Figure 1). The subjects were randomly assigned to either one of the test groups (Group A: *L. paracasei*, Group C: *L. plantarum*) or the placebo group (Group B).

The study comprised a screening visit and six study visits. Before each study visit, subjects were instructed not to eat or drink (with the exception of water) 2 h before the appointment and to refrain from oral hygiene for 24 h (except for visits 4 and 5 which are the experimental gingivitis period).

The subjects started with the wash-in period of 2 weeks – the intervention (the use of one of the two oral probiotics or a placebo) – on day zero (visit 1) of the study, while continuing their regular oral hygiene measures (Figure 1, Supplementary Figure 1). After one (visit 2) and 2 weeks (visit 3), samples were collected for assessment of the dynamics of the biochemical, immunological and microbiological aspects of the oral ecosystem and at the end of the wash-in period (visit 3) – plaque and gingival health were assessed. After the wash-in period, the experimental gingivitis period followed during which the subjects continued to use their assigned lozenges three times daily. The subjects were requested to refrain from any form of oral hygiene for 2 weeks. After one (visit 4) and 2 weeks (visit 5), the subjects visited the dental clinic for a research appointment. After the experimental gingivitis period, the subjects stopped using the intervention (probiotic or placebo) and restarted their routine oral hygiene. After 2 weeks of this wash-out period, the last research visit (visit 6) was scheduled for assessment of the dental plaque and gingival health and for sample collection.

### Sample Size Calculation

The sample size was calculated *a priori* with G^*^Power [G^*^Power 3.1.9.2, Faul, Universitaet Kiel, Germany [15]]. There is no literature available on the effect of the specific probiotic test strains on oral health using bleeding on marginal probing (BOMP) as the primary parameter. The sample size calculation was therefore based on the estimation that the expected effect will be between small and medium [Cohen d between 0.2 (small) and 0.5 (medium) using parametric analysis of variance] since probiotics are not expected to have major effects. Thus, the effect size was set at d = 0.35. The *p*-value was set at 5% (probability of a type I error, false-positive rate) and the power at 80%. This resulted in a total sample size of 84. To account for potential dropouts during the study, 20% additional subjects [16] were added to the sample size calculations. In case non-parametric testing becomes necessary, the sample size was increased by an additional 15% [17]. This led to a final sample size of 117 with 39 subjects per group.

### Randomization, Blinding, and Interventions

The study subjects were allotted a unique subject identifier consisting of 4 random numbers after the screening visit. Before the start of the study, each subject identifier was linked to one of the two test groups or the placebo group using computer-generated stratified block randomization.

This study was triple blinded: the subject, the investigators, and the outcome assessors were blind to the treatment allocation. Randomization into three equal-sized groups was performed by a senior researcher not involved in the clinical part of the study. Random allocation of the three groups to one of the two test lozenges or the control lozenge was provided by the subsidizing party and was kept in a sealed file with the sponsor of the study. The treatment frequency, duration, and amount of food ingredient (lozenges) were identical in all three groups: 1 lozenge 3 times daily (after each meal), for 4 weeks. The lozenges were distributed to subjects in blister packs pre-labelled with the subject identifier by the subsidizing party. The subjects were instructed to dissolve the lozenges in the mouth without chewing or swallowing and refrain from oral hygiene and from rinsing their mouth for the first 30 min after using the lozenge.

The test products contained microcrystalline cellulose, mannitol, silicon dioxide, crosslinked polyvinylpyrrolidone, carbomer, sodium stearyl fumarate, flavor, acrylates copolymer, and freeze-dried *L. paracasei* LPc- G110 [CCTCC M 2013691; BioGrowing Co., Ltd., Lactobacillus paracasei LPc-G110 (CCTCC M 2013691), Qingpu Industrial Zone Shanghai, P.R. China], or *L. plantarum* GOS42 [DSM 32131; Probi AB, Lactobacillus plantarum GOS42 (DSM 32131), Ideon Gamma 1, Lund, Sweden]. In the placebo lozenges, the use of a dye (Symcolor Eisenoxidgelb E172; Symrise AG PN 656835) was used to match the color of the test lozenges. The test lozenges contained the probiotics in the following dosages: 17.6% of *L. paracasei* (LPc-G110) corresponding to 35.2 mg LPc-G110 per lozenge, at least 1 x 10^9^ CFU/lozenge, or 10.1% of *Lactobacillus plantarum* (GOS42) corresponding to 20.2 mg GOS42 per lozenge, at least 5 x 10^9^ CFU/lozenge. The lozenges were stored at 2–8°C and tracked under temperature control (2–8°C) during the shipment to the study site.

Upon arrival at ACTA, the blister packs containing the test and placebo lozenges were stored at 2–8°C until distribution to the participants. The participants were asked to store the lozenges at room temperature.

### Procedures During the Study Visits

A schematic overview of the study procedures during the study visits is presented in Supplementary Figure 1. At each study visit, first, three fluorescence and white light photographs were taken to assess the red fluorescence of plaques (RFP) [18, 19]. This was done on the vestibular aspects of the front and lateral teeth using a Canon 450-D SLR camera equipped with a Biluminator tube (QLF-D system, Inspektor Research Systems BV, Amsterdam, the Netherlands) and cheek retractors (Henry Schein, Gillingham, UK) *via* image capture software on the PC (C3 1.25 Inspektor Research Systems BV, Amsterdam, the Netherlands). The percentage of RFP coverage was calculated using dedicated RFP analysis software (QA2, v1.27, Inspektor Research Systems BV., Amsterdam, the Netherlands), and the average RPF of the three images (front, left, and right) per visit was calculated.

Stimulated saliva samples were collected at each visit by chewing a gum base and drooling saliva into a sterile 50-ml polypropylene tube (Sarstedt Ltd., Numbrecht, Germany) while holding it on ice for 5 min. Right thereafter, the samples were vortexed and aliquoted in two sterile 2-ml Eppendorf tubes, one for microbial and the other for immunological analyses, snap-frozen in ethanol on dry ice, and stored at −80°C.

On visits 1, 3, 5, and 6, plaque index was determined by a calibrated dental hygienist (Cronbach's alpha 0.844) at two predetermined quadrants (either 1^st^ and 3^rd^ or 2^nd^ and 4^th^ quadrant) of the mouth. The choice of the quadrants was determined by throwing a dice at the first study visit. Six sites per tooth were scored in two quadrants: the disto-vestibular, mid-vestibular, mesio-vestibular, disto-lingual, mid-lingual, and the mesio-lingual sites from 0 (no plaque), 1 (a thin film of plaque which cannot be seen with the naked eye, but assessed by using a probe), 2 (moderate plaque accumulation) to 3 (abundant accumulation of plaque) according to the study conducted by Silness and Löe [20]. Plaque index (PI) was calculated per participant: the assessed scores per site were totaled and divided by the number of sites assessed. PI% was calculated by dichotomized use of plaque scores as in absence or presence of plaque. Plaque scores 1-2-3 were recoded into score 1, and the total number of sites with score 1 was divided by the total number of sites assessed and then multiplied by 100 to create a percentage score.

Thereafter, at each study visit, a tongue swab and supragingival plaque were collected for microbiological analyses as described previously [21]. In brief, the tongue sample was collected by striking four strikes with a sterile microbrush (Microbrush International, Grafton, USA) over the dorsum of the tongue in a longitudinal direction. The tip of the brush with the sample was cut into a sterile Eppendorf vial. A supragingival plaque sample was collected from buccal surfaces of the first and the second upper molars, contralateral to the quadrant where plaque score was determined, using a sterile plastic spatula (KerrHawe, Bioggio, Switzerland). The sample was scraped into the lid of a sterile Eppendorf vial, the vial closed and centrifuged for 1 min at 14,000 rpm. Both samples were put on ice for a maximum of 2 h and stored at −80°C until further processing for 16S rDNA amplicon sequencing.

On visits 1 and 3, an additional supragingival plaque sample was collected in the same way as above but from the lingual surfaces of the lower first and second molars, for extraoral plaque pH measurements. The sample was immediately transported to the laboratory and processed at room temperature by vortexing with 50 μL of sterile saline and determining pH using a calibrated pH electrode (Sentron, Leek, the Netherlands).

At visits 1, 3, 5, and 6, the Modified Gingival Index (MGI) [22] and the BOMP [23, 24] were measured. MGI is recognized as a non-invasive index for the measurement of absence or presence of color change in the gingivae and assesses the extent and severity of the inflammatory change (based on color, texture, volume, and extent) visually. The MGI index is scored using an ordinal five-point scale (0–4), which represents a ranking order from the absence of any visible sign of inflammation (Score 0) to mild (Score 1), moderate (Score 2), and severe inflammation (Score 3) or severe inflammation with spontaneous bleeding, congestion, or ulceration (Score 4). This score was given in two preselected quadrants to six gingival areas per tooth: the disto-vestibular, mid-vestibular, mesio-vestibular, disto-lingual, mid-lingual, and mesio-lingual sites. The MGI index and MGI% were calculated according to the same procedure as described for plaque. For BOMP, a bleeding score was given in the same two preselected quadrants to six gingival areas of the tooth. The index has a three-point scale (0–2) to describe the bleeding tendency on the buccal or lingual aspects of each tooth (0 – no bleeding; 1 – pin-prick bleeding; 2 – excessive bleeding). The BOMP score and BOMP% were calculated according to the same procedure as for plaque and MGI.

At visit 2, the current and past dental caries experience was assessed as several decayed, missing, or filled surfaces (DMFS), according to the WHO criteria [25] on all teeth but third molars.

#### Product Tolerance

At visits 2, 3, 4, and 5 the subjects were asked to rate their experience regarding the lozenges, at a scale between 0 (very unpleasant) and 10 (very pleasant).

#### Compliance

Compliance was defined as % compliance = (number of lozenges taken / number of lozenges supposed to have been taken) ^*^ 100%, based on the number of the returned lozenges and the number of expected lozenges to be taken.

### Sample Processing and Analyses

#### Inflammatory Markers in Saliva

The pro-inflammatory cytokines interleukin 1 ß (IL-1β) and prostaglandin 2 (PGE2) were selected to be measured in the clinical samples based on previous studies where the periodontal disease was associated with a raise of IL-1ß and PGE2 [26, 27]. Concentrations of IL-1β and PGE2 in saliva samples were determined by enzyme immunoassay for IL-1ß (#DY201, Range: 3.9–250 pg/ml; Bio Techne/R&D Systems; Minneapolis, USA) and for PGE2 (#514010, Range: 7.8–1,000 pg/ml; Cayman; Michigan, USA) respectively, according to the manufacturer's instructions. Saliva samples were thawed, centrifuged for 5 min at 13,400 rpm, and the supernatant either diluted 1/5 (PGE2) or 1/10 (IL-1 ß) with the respective assay buffer. The assays were performed in duplicate with all samples per individual subject on a single ELISA plate. In order to monitor inter-assay variability and repeatability, an internal control standard containing saliva of employers of VivaCell was established and run on each sample carrier as sextuplicate.

#### Bacterial DNA Isolation, Quantification, and Sequencing

Plaque, tongue and saliva samples were subjected to DNA isolation in batches of 84 samples per sample type with all samples of each subject included in the same batch. Several types of controls were included at critical steps in the process to trace possible inter-batch and intra-sample variations: blank isolations, repeated control isolations (aliquoted stimulated saliva from a co-worker, isolated with each DNA isolation batch), negative PCR blanks, a known mock community sample (positive control; BEI resources, cat. HM-782 of genomic DNA from 20 bacterial isolates) and Run to Run controls (three samples from the first batch of isolated samples). All designated controls were included in the final amplicon mixes and sequenced. For each sample type, the samples were divided over two final amplicon mixes and run on the Illumina Miseq platform (the MiSeq platform is of Illumina (R) Inc., San Diego, CA, USA: https://emea.illumina.com/systems/sequencing-platforms/miseq.html) in 6 sequencing runs.

For plaque analysis, the vials were thawed, and the plaque was resuspended with 150 μL of sterile Tris-EDTA buffer (TE buffer) and transferred to an assigned well in a 1.1 ml deep-well plate. For stimulated saliva, the vials were thawed and vortexed extensively and 150 μl of saliva was transferred to an assigned well. For tongue coating, the vials were thawed and the microbrushes were transferred to an assigned well, using sterile forceps, then 150 μl of TE buffer was added to pick up possible leftovers. After resuspending the possible contents, the fluid was transferred to the same well as the microbrush. The 1.1 ml deep well plate (Axygen Scientific Inc., CA, USA), contained 250 μl 0.1-mm Zirconia beads, 200 μl of phenol (Rotiphenol, Carl Roth GMBH&Co. KG, Karlsruhe, Germany), and 200 μl of lysis buffer (MagMini DNA isolation kit, LGC Genomics Ltd, Middlesex, UK). The deep well plate was sealed and subjected to 4 times 2 min of bead-beating in a Mini-BeadBeater-96 (BioSpec Products, Bartlesville, OK, USA). DNA was extracted and purified using the MagMini DNA Isolation Kit (MagMini DNA isolation kit, LGC Genomics Ltd, Middlesex, UK).

Bacterial DNA concentration was determined by quantitative PCR, with universal primers specific to the bacterial 16S rRNA gene (forward: TCCTACGGGAGGCAGCAGT; reverse: GGACTACCAGGGTATCTAATCCTGTT; probe: 6FAM-CGTATTACCGCGGCTGCTGGCAC-BHQ1) [28] using E. coli K12 DNA for standard curves.

Sample DNA was diluted to 200 pg/μL with PCR-grade water and the V4 hypervariable region of the 16S rRNA gene was amplified using 1 ng DNA with 1 μM of each primer and 30 amplification cycles [29]. PCR products were subjected to AMPure XP (Agencourt AMPure xP, Beckman Coulter, Nyon, Switzerland) clean-up to remove primers dimers and were assessed for bands with agarose gel electrophoresis. Samples with no clear reason for failing PCR were redone. The PCR yield was determined with picogreen fluorescent dye (QUANT-IT, Invitrogen; Thermo Fisher Scientific Inc., Landsmeer, the Netherlands) and samples were equimolarly pooled with 200 ng DNA per sample. For samples with low yield and blanks, a fixed amount of μL product was added. Paired-end sequencing (2 × 251 nucleotides) of the DNA was conducted on the Illumina MiSeq platform at the AUMC Cancer Center Amsterdam (Amsterdam, the Netherlands). The flow cell was loaded with 12 pmol DNA including 25% PhiX.

#### Determination of the Fungal DNA Concentration

Concentration of fungal DNA in plaque was determined on DNA isolated from dental plaque as described above with RT-PCR using primers for the fungal 28S rRNA gene (forward: GCATATCAATAAGCGGAGGAAAAG; reverse: TTAGCTTTAGATGATTTACCACC; probe: CGGCGAGTGAAGCGGSAARAGCTC) [30]. The fungal load was calculated relative to the bacterial (16S rRNA gene) DNA amount per sample.

### Species-Specific Quantification of the Test Strains by qPCR

The bacterial strains used for qPCR quantification in this study were *Lactobacillus paracasei* subsp. *paracasei* DSM 5622, *Lactobacillus plantarum* DSM 20174, and *Pediococcus acidilactici* DSM20284 (negative control) obtained from the German Collection of Microorganisms and Cell Cultures GmbH (DSMZ, Braunschweig, Germany). qPCR of these species was performed to verify that these probiotic strains were present in the samples and to determine their quantity.

For the creation of standard curves, the two types of strains of *Lactobacillus paracasei* subsp. *paracasei* DSM 5622 and *Lactobacillus plantarum* DSM 20174 were inoculated from cryotubes by thawing the tubes at 37°C for 5 min and transferring 1 ml culture to 19 ml of sterile, pre-warmed MRS broth medium (Oxoid, Basingstoke, UK). Cells were cultured overnight at 37°C with agitation at 100 rpm under aerobic conditions. DNA was extracted from these overnight cultures using the Mag™ Mini Kit (LGC Biosearch Technologies Ltd., UK) following the manufacturer's protocol with the following deviations: 1 ml of overnight culture was centrifuged at 14,000 × g, the supernatant was discarded, and the pellet was resuspended in 100 μl of 50 mM EDTA, and the final elution step, using 250 μl of elution buffer. After 3 min of separation via magnetic force, 200 μl were transferred to a fresh microcentrifuge tube.

Quality control was performed by agarose-gel electrophoresis. Purity was assessed based on its absorbance and elucidation of gDNA concentrations. Measurement of absorption ratios (A_260nm_/A_280nm_ and A_260nm_/A_230nm_) was performed with BioDrop Duo+ photometer (Biochrom Ltd., UK).

Measurement of gDNA concentration was performed using Invitrogen Quant-iT™ dsDNA Assay Kit on a FLUOstar Omega (BMG Labtech, Ortenberg, Germany) following the manufacturer's protocol. A new standard curve was prepared for each determination.

Primers and probes specific for *L. paracasei* followed the design described by Byun et al. [31] with a single deviation in the reverse primer selecting a cytosine nucleotide as pyrimidine base (Supplementary Table 1). Oligonucleotides for specific detection of *L. paracasei* target variable regions V1 and V2 of 16S rRNA gene.

Primers and probes specific for *L. plantarum* were constructed to target at domain II in the 23S rRNA gene. In brief, primers were designed by pairwise alignment using CLC sequence viewer 8 (Qiagen, Hilden, Germany) with standard parameters (Gap open cost 10, Gap extension cost 1, End gap cost: as any other, Alignment: very accurate) in a first step for non-homologous sequences in the order of *Lactobacillales* followed by a second manual selection of suitable sequences within the family of *Lactobacillaceae* as indicated below (Deposit; GenBank accession numbers or NCBI Nucleotide database in parentheses): *Aerococcaceae* (Tax-ID: 186827): *Abiotrophia defectiva* [ATCC 49176, ACIN03000005.1 (Region 146250-148775)], *Aerococcus christensenii* [DSM 15819, NZ_CP014159 (Region 238537-241445)], *Aerococcus sanguinicola* [DSM 15633, NZ_CP014160.1 (Region 192032-194939)], *Dolosicoccus paucivorans* (DSM 15742, FNEL01000099.1), *Facklamia miroungae* (ATCC BAA-466, FNCK01000021.1), *Ignavigranium ruoffiae* (DSM 15695 FOEN01000030.1); *Carnobacteriaceae* (Taxonomy ID: 186828): *Alkalibacterium gilvum* (DSM 25751, FNYW01000080.1), *Atopostipes suicloacalis* (DSM 15692, FQUF01000049.1), *Carnobacteriuminhibens ssp. Gilichinskyi* strain WN1359T [DSM27470T, NC_022606.1 (Region 463871-466793)], *Desemzia incerta* (DSM 20581, FOXW01000018.1), *Granulicatella adiacens* (DSM 9848, ACKZ01000001.1), *Isobaculum melis* (DSM 13760, FOHA01000042.1), *Marinilactibacillus psychrotolerans* (DSM 19582, FMDZ0100041.1); *Enterococcaceae* (Taxonomy ID: 81852): *Enterococcus cecorum* strain SA3 [NZ_CP010064.1 (Region 1391193-1394107)], *Melisococcus plutonius* [ATCC 35311, NC_015516.1 (Region 1405070-1407978)], Vagococcus sp. AM17-17 (QRJY01000033.1); *Lactobacillaceae* (Taxonomy ID: 33958): *Lactobacillus delbrueckii ssp. Bulgaricus* [ATCC 11842, NC_008054.1 (Region 691149-694059)], *Lactobacillus paracasei ssp. Paracasei* [DSM 5622, NZ_AP012541.1 (Region 799668-802587)], *Lactobacillus plantarum* (DSM 20174, BALV01000030.1), *Lactobacillus fermentum* [DSM 20052, ACGI010000131.1 (Region 131-3049)], *Lactobacillus salivarius* [DSM 20555, ACGT01000024.1 (Region 139-3052)], *Lactobacillus crispatus* ST1 [NC_014106.1 (Region 429915-432826)], *Lactobacillus gasseri* [DSM 20243, NC_008530.1 (Region 1576872-1579779)], *Lactobacillus rhamnosus* [DSM 14870, NC_CP006804.1 (Region 643619-646539)], *Lactobacillus reuteri* [DSM 20016, NC_009513.1 (Region 179497-182423)], *Pediococcus acidilactici* [DSM 20284, NZ_CP015206.1 (Region 633234-636160)], *Leuconostocaceae* (Taxonomy ID: 81850): *Fructobacillus durionis* [DSM 19113, FOLI01000015.1 (Region 203-3110)], *Oenococcus kitaharae* [DSM 17330, NZ_CM001398 (Region 1065792-1068681)], *Leuconostoc citreum* [DSM 5577, NC_010471.1 (Region 434873-437792)], *Weissella bombi* [DSM 28794, partial: FMAO010000003.1 (Region 129210-129516)], *Streptococcaceae* (Taxonomy ID: 1300): *Lactococcus chungangensis* (DSM 22330, FPKS01000037.1), *Streptococcus mutans* [DSM 20523, NZ_LS483349.1 (Region 415939-418843)], *Streptococcus ratti* [DSM 20564, AJTZ01000001.1 (Region 176-3076)]; Others (Outgroup): *Tannerella forsythia* 92A2 [CP003191.1 (Region 686088-688916)], *Porphyromonas gingivalis* [DSM 20709, NC_010729.1 (Region 479572-482470)], and *Filifactor alocis* [ATCC 35896, NC_016630.1 (Region 716154-719059)].

Binding sequences, amplicon sizes, and melting temperatures are shown in Supplementary Table 1. Each probe displays 5' FAM fluorescence (fluorescein) accompanied by a black hole quencher (BHQ-1) at 3' prime.

Specificity of primers/probes was verified by *in silico* analysis using NCBI Basic Local Alignment Search Tools (Taxonomy: ID 186826, Database: representative genomes) as well as by *in vitro* analysis against type strains of different genera belonging to the order of *Lactobacillales*.

For assessment of bacterial quantity in clinical samples, calibration curves are based on type strains *L. paracasei* subsp. *paracasei* DSM 5622 and *L. plantarum* DSM 20174 were constructed. Standard curves were serially 10-fold diluted up to 6 dilution steps displaying a range of 5 × 10^6^ target copies to 5 target copies per reaction.

Prior experiments assured a good correlation for readout of qPCR results and bacterial numbers based on colony-forming units (data not shown). Based on the DNA concentration and the genome size of the type strains, the target gene copy number per reaction was calculated accordingly and corrected by the operon number, i.e., the number of gene copy numbers per genome. This provided an estimation of bacterial load based on genomic equivalents and allowed for linear regression based on C_q_-values of samples:


$$\begin{array}{l} \text{Target copy number per reaction}=\frac{c_{gDNA}*N_{A}}{N^{*}A_{bp}*10^{9}\frac{ng}{g}} \end{array}$$

C_gDNA_ = Concentration of gDNA [ng/μL]

N_A_= Avogadro's number 6,022 ^*^ 10^23^ molecules/ mole

N = Genome size [bp]

A_bP_= Average mass of 1 basepair ds DNA [660g / mole of bp].

All clinical samples were run on a Roche LightCycler® 480 System (Roche, Basel, Switzerland) and the LightCycler® 480 software release 1.5.1.62 (Roche, Basel, Switzerland) with the matching master mix (Light Cycler® 480 Probes Master, #04902343001, Roche, Basel, Switzerland) and PCR grade water. Cycling conditions for the quantification of the investigational product were applied as follows: initial pre-incubation at 95°C for 10 min (ramp speed 4.4°C/s) followed by 45 cycles with a sequence of 95°C 15 s (ramp speed 4.4°C/s), 63°C for 60 s (ramp speed 2.2°C/s) and a final cooling step of 40°C for 10 s (ramp speed 1.5°C/s).

The total reaction volume was 20 μL with 2 μL of sample or DNA-standard as a template. For *L. plantarum*, the following concentrations of primers were used: 800 nM forward primer (Lplan-F), 800 nM reverse primer (Lplan-R), and 200 nM probe (Lplan-P). For *L. paracasei*, the following concentrations of primers were used: 700 nM forward primer (Lpara-F), 700 nM reverse primer (Lpara-R), and 200 nM probe (Lpara-P). All working steps have been performed with DNA-free, DNase-free, RNase-free, pyrogen-free, PCR-inhibitor-free, and sterile pipette tips.

*Lactobacillus paracasei* and *L. plantarum* qPCR was performed on the samples of all participants from three different sites (plaque, dorsum of the tongue, and saliva) collected at visits 1, 3, 5, and 6. All samples were pipetted as technical duplicates in 96-well qPCR plates (VWR, Darmstadt, Germany) and covered with qPCR Optical Seals (Eurogentec, Seraing, Belgium). Each reaction plate carried a full range of samples collected per subject from a single sampling site, accumulating up to 8 subjects per plate. Each plate contained standard, negative (DNA of *Pediococcus acidilactici* DSM 20284), and positive control samples (DNA of *L. paracasei* LPc-G110 or DNA of *L. plantarum* GOS 42), and a non-template control with PCR grade water instead of DNA. Plates were centrifuged at 2,000 × g for 2 min before performing qPCR analysis.

### Sequencing Data Processing

All paired-end reads were processed together. The processing was described in detail in a previous study [32]. Below, all steps are summarized. First, the paired-end reads were merged and quality-filtered using USEARCH [33, 34]. Next, the merged reads were error-corrected using UNOISE3 [35], and the (quality-filtered) sequences were mapped to the centroids produced by UNOISE3 to construct a zOTU table. Taxonomic names were assigned to the most abundant sequence of each zOTU using the human oral microbiome database HOMD version 14.51 [36]. The taxonomic assignments were carried out using QIIME version 1.8 [37] and the RDP classifier [38] (min. confidence 0.8). Since the use of a region-specific taxonomic database improves taxonomic assignments [39, 40], the HOMD 16rRNA RefSeq alignment was first trimmed to the V4 region. Then, the V4-specific alignment was converted to a set of non-redundant sequences which was used to retrain the RDP classifier. The final zOTU table, including the taxonomic assignments, was subsampled at 8,000 reads per sample to allow comparisons among samples with different sequencing depths.

### Study Endpoints

#### The Main Study Endpoint

- The change in gingival bleeding on marginal probing (ΔBOMP index) between the end of the experimental gingivitis period (V5) and the baseline visit (V1). The BOMP index accounts for the severity of the inflammation.

#### Secondary Study Endpoints

- The change in the severity of gingival bleeding (BOMP) between the end of the wash-in period (V3) and the baseline visit (V1), and between the end of the wash-out period (V6) and V1.
- The change in the percentage of the bleeding index indicates the change in the prevalence of gingival inflammation: (A) between the end of the wash-in period (V3) and the baseline (V1); (B) between the end of the experimental gingivitis period (V5) and V1 and (C) between the end of the wash-out period (V6) and V1.
- The changes in the concentration of inflammatory markers PGE2 and IL1-ß in saliva: (A) between the end of the wash-in period (V3) and the baseline (V1); (B) between the end of the experimental gingivitis period (V5) and V1; and (C) between the end of the wash-out period (V6) and V1.
- Changes in bacterial concentration, bacterial profiles and diversity, and in fungal abundance between V1 and V2, V1 and V3, V1 and V4, V1 and V5, and V1 and V6.

#### Other Study Endpoints

1. Changes in the MG:

The change in the severity of the inflammation (ΔMGI) was assessed: (A) between the end of the wash-in period (V3) and the baseline (V1); (B) between the end of the experimental gingivitis period (V5) and V1; and (C) between the end of the wash-out period (V6) and V1.

The change in the percentage of the sites with gingival inflammation, ΔMGI%, was assessed: (A) between the end of the wash-in period (V3) and the baseline (V1); (B) between the end of the experimental gingivitis period (V5) and V1; and (C) between the end of the wash-out period (V6) and V1.

2. Change in the PI:

The change in the abundance (ΔPI) of the dental plaque was assessed: (A) between the end of the wash-in period (V3) and the baseline (V1); (B) between the end of the experimental gingivitis period (V5) and V1 and (C) between the end of the wash-out period (V6) and V1.

The change in the percentage of the sites with dental plaque, ΔPI%, was assessed: (A) between the end of the wash-in period (V3) and the baseline (V1); (B) between the end of the experimental gingivitis period (V5) and V1; and (C) between the end of the wash-out period (V6) and V1.

3. Change in the RFP:

The change in the average coverage percentage of red fluorescent dental plaque (ΔRFP%) was calculated between V1 and V2, V1 and V3, V1 and V4, V1 and V5, and V1 and V6.

4. Change in the resting pH of the supragingival plaque between visits 1 and 3.

### Protocol Deviations

The following were pre-defined as *major protocol deviations*:

- The subject brushed teeth during the experimental gingivitis period.
- The subject did not brush the teeth during the wash-in period.
- The subject used probiotic products other than the investigational product.
- The subject's missed visit(s).
- Poor compliance: subject did not use the study product for at least 80% of the expected use.

The following protocol deviations, *minor* for the primary outcome, were considered detrimental for the following secondary and additional outcomes:

- Plaque pH:
  ° The subject had food or drink intake (other than water) less than 2 h ago.
  ° The subject had brushed his/her teeth less than 16 h ago.
- RFP images of dental plaque:
  ° The subject had brushed his/her teeth less than 16 h ago.
  ° Measurements potentially disturbing dental plaque (e.g., plaque sampling, BOMP) had been performed before the RFP images were taken.
- Plaque microbiological composition:
  ° The subject had brushed his/her teeth less than 16 h ago.
- Plaque index:

The subject had brushed his/her teeth less than 16 h ago. The respective values were regarded as invalid and were excluded from the per-protocol (PP) analyses for the respective secondary/additional outcome.

### Statistical Methods

Statistical analysis was performed blind to the test and placebo group allocation, after the data review and assessment of analysis populations. The statistical analysis plan was reviewed and approved by an independent statistician (C. Baljé, AUTHOR! et al. BV, The Netherlands; https://www.author.nl/). The safety analysis (SA) population was used for the safety analysis. The PP population was used for the efficacy analysis. All primary and secondary outcomes were compared among the three study groups. The normality of the data was tested using the Shapiro-Wilk test. With normal data distribution and equal homogeneity of variances (Levene's test of variance), the one-way ANOVA was used, followed by Tukey *post-hoc* test. If data normality was not confirmed, data were log-transformed and the normality analysis repeated. In case of a significant outcome, the Kruskall-Wallis test was used with *post-hoc* Mann-Whitney U tests to identify the differences. The *p*-values were corrected for multiplicity using a False Discovery Rate (FDR) correction. Categorical variables (e.g., toothbrush type, tongue brushing habit) were compared among the groups using Chi-square tests.

Next to the primary and secondary analyses, ancillary analyses were performed to assess changes in various output variables in time, within each group. For this, the General Linear Model Repeated Measures test, followed by Paired samples *t*-test, was performed in cases with normal data distribution, while the Friedman test, followed by Wilcoxon Signed Ranks test, was performed with not normally distributed data. No corrections for multiple comparisons were performed on the obtained *p*-values.

Principal component analysis (PCA) was performed on subsampled and log-2 transformed microbial profile data. The similarity in microbiome profiles was assessed using Bray-Curtis similarity distance. Alpha diversity was assessed using the Shannon Diversity Index and species richness (nr of zOTUs/sample). All analyses above were performed using PAST software version 3.18 [41]. Differences in microbial profiles among the sample groups (beta diversity) were assessed with one-way permutational multivariate ANOVA (PERMANOVA) using the Bray-Curtis similarity measure. For comparisons of microbiome profiles of related samples, PERMANOVA with restricted permutations by the individual subjects was performed. All PERMANOVA analyses were performed using adonis2 (R version 3.6.1 ([42]). *P*-values were corrected for multiple testing using FDR correction.

## Results

### The Screening and Disposition of Subjects

**Figure 2:**
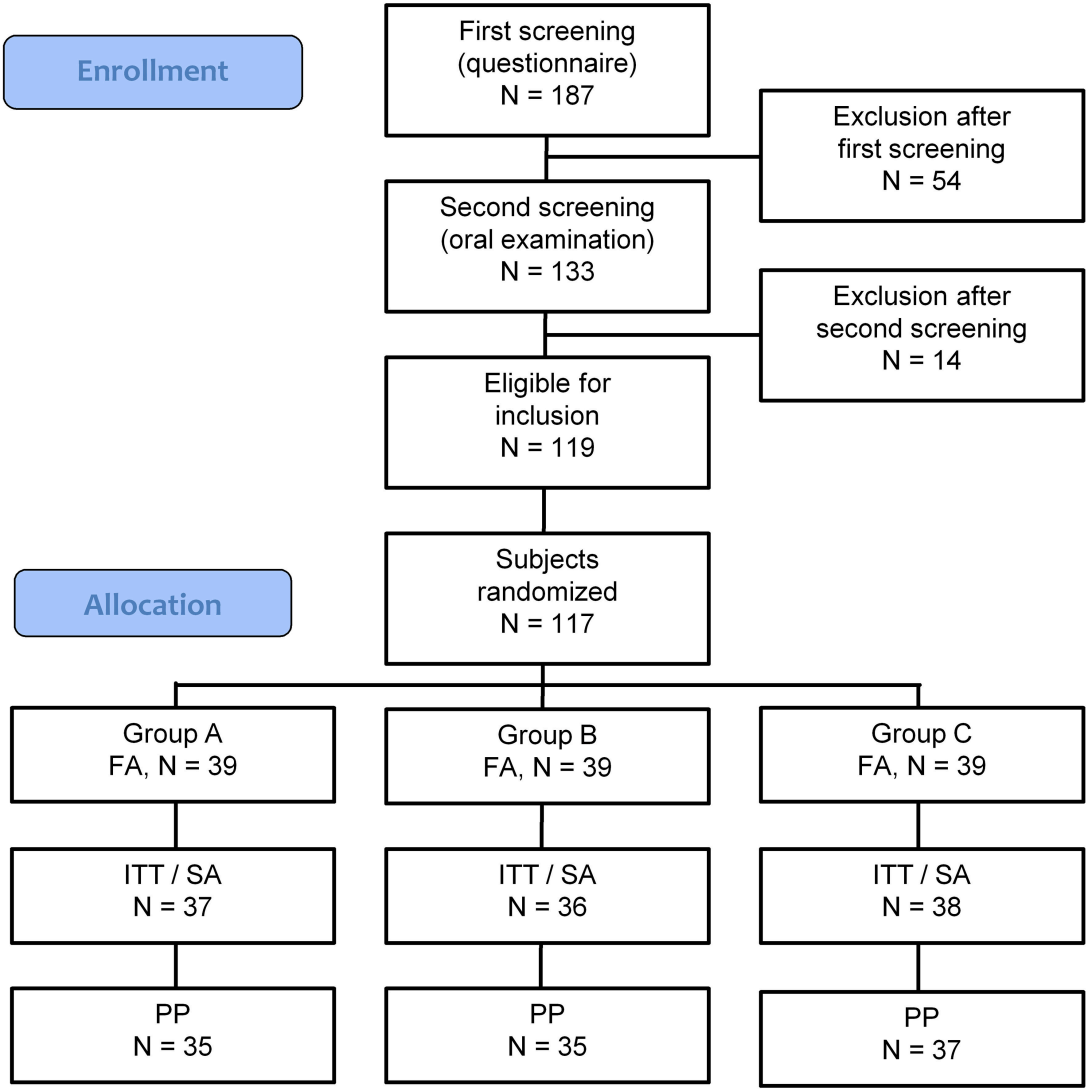
CONSORT-flow chart showing the disposition of subjects. FA, Full Analysis population; ITT, Intention to Treat population; SA, Safety Analysis population; PP, Per Protocol population. Group A: *Lactobacillus paracasei* LPc-G110 containing lozenges, Group B: placebo lozenges, Group C: *Lactobacillus plantarum* GOS42 containing lozenges.

The screening of potential subjects was executed between April 18 and May 9, 2018. In total, 187 potential subjects were screened using a questionnaire. Among them, 54 (28.9%) were excluded due to various reasons (Supplementary Table 2). The remaining 133 potential subjects received a clinical examination, of which 119 were found eligible to participate in the study (Supplementary Table 3). Two included participants were regarded as a backup if there would be unexpected dropouts before the randomization of the study subjects, which was not the case, and were thus excluded from the study, resulting in the required 117 participants (Figure 2).

### Protocol Deviations

The full analysis (FA) set included 117 subjects, while the intention to treat (ITT)/SA set contained 111 subjects. Of the six study dropouts, two subjects did attend the first visit and did not provide a reason for withdrawal, while one subject reported pregnancy (exclusion criterium), one had to continue the use of the orthodontic night guard and did not meet the inclusion criteria anymore, while two subjects did not attend all scheduled visits anymore due to changes in their private situation.

Among the 111 subjects, four cases of major protocol deviations were observed during the study, leading to 107 subjects in the per-protocol (PP) analysis set. Two of the four individuals stopped participating in the study for personal reasons after 1 and 2 weeks, respectively, while two individuals misunderstood the study protocol and abstained from toothbrushing before the actual experimental gingivitis phase started.

In total, 89 minor protocol deviations were observed during the study (Supplementary Table 4). The majority of the minor deviations (72 of 89) accounted for missed intake of the lozenge(s). Group B had the highest number of reports of missed intake (34 reports), while groups A and C had 17 and 21 reports, respectively. Treatment group C had a higher frequency of minor protocol deviations (*n* = 5), which had consequences for secondary output parameters (plaque pH, plaque composition, and/or red plaque fluorescence) than groups A or B (*n* = 1 for each).

### Treatment Compliance

The study visits comprised the period between May 16th and June 29th, 2018. At the end of the study, subjects were asked to return the unused lozenges. The compliance was calculated as a proportion of the lozenges taken based on the number of the returned lozenges over the estimated number of the lozenges to be taken. To allow for unintentional loss of lozenges, more lozenges were dispensed than required for the study duration to the participants. Average compliance in the PP population in group A was 98% (*SD* 5.5), in group B was 99% (*SD* 5.4), and in group C was 101% (*SD* 9), where one individual did not return any lozenges, resulting in a 135% compliance.

### Adverse Events

In total, 41 adverse events (AEs) were reported during the study (Supplementary Table 5). Of these, 34 were reported as mild, 7 – as moderate, and none – as serious adverse events. At visits 1 and 6 there were no AEs reported. All 41 AEs were registered from visit 2 until visit 5, thus during the exposure to the study products. Among the 41 AEs, 18 were classified as possibly related to the treatment. The majority of these were aphthae (11 events in 9 individuals) or sensitive teeth (6 events in 3 individuals). Sensitive teeth were observed exclusively in group B and were reported at visits 4 and 5 (experimental gingivitis period). Most complaints of aphthae were reported in groups A and C. In group A, aphthae were reported at visits 3, 4, and 5, in group B – at visit 5, and in group C – at visits 2 and 5. A single event of stomach cramps was reported in group C at visit 4.

### Product Tolerance

At each visit that fell within or followed the intervention period, the subjects were asked to rate their experience regarding the lozenges, on a scale ranging from 0 (very unpleasant) to 10 (very pleasant). In all groups, the lozenges were well tolerated, with a few exceptions in the first study weeks in group B (Supplementary Table 6).

### Baseline Data

Baseline characteristics of the study subjects in the ITT/SA population (*n* = 111) are shown in Table 1. Both age and DMFS were not normally distributed (*p* < 0.05, Shapiro-Wilk test) and were therefore tested non-parametrically. There were no significant differences found among the groups by age and DMFS (*p* > 0.05, Kruskal-Wallis test), nor by other parameters determined at baseline: gender, preferred brushing hand, the type of toothbrush used, the toothbrushing frequency, tongue brushing habit, or the use of mouthwash before the study (*p* > 0.05, Chi-square test). Among the clinical parameters, there was a borderline difference found in BOMP among the study groups (*p* = 0.05, Kruskal-Wallis test), while for the microbiological parameters a significant difference was found among the study groups in *L. paracasei* genomic units in the tongue samples (*p* = 0.02, Kruskal-Wallis test): samples from group B had significantly less *L. paracasei* than samples from group A (*p* = 0.033, Mann-Whitney test, FDR corrected) or group C (*p* = 0.0167).

**Table 1:** Baseline characteristics of the study subjects [safety analysis (SA)/ intention to treat (ITT) population].

		**Group A** **(*N =* 37)**	**Group B** **(*N =* 36)**	**Group C** **(*N =* 38)**
Gender	Male	11 (30%)	11 (31%)	13 (34%)
	Female	26 (70%)	25 (69%)	25 (66%)
Age (years)	Mean (SD)	26.5 (7.9)	24.8 (7.4)	25.8 (8.0)
	Median (Range)	24 (18–50)	24 (18–53)	23 (18–51)
DMFS	Mean (SD) 95% CI Median (Range)	5.0 (6.8) 2.8–7.3 2 (0–28)	3.7 (6.0) 1.7–5.7 2 (0–26)	4.3 (7.0) 2–6.6 0.5 (0–27)
Favorite brushing hand	Right Left Both	30 (81%) 7 (19%) 0	30 (83%) 5 (14%) 1 (3%)	35 (92%) 3 (8%) 0
Toothbrush	Manual	16 (43%)	18 (50%)	21 (55%)
	Electric	14 (38%)	8 (22%)	14 (37%)
	Both	7 (19%)	10 (28%)	3 (8%)
Toothbrushing times/day	Once Twice More often	7 (19%) 28 (76%) 2 (5%)	5 (14%) 30 (83%) 1 (3%)	7 (18%) 31 (82%) 0
Regular tongue brusher	No Yes	27 (73%) 10 (27%)	18 (50%) 18 (50%)	23 (61%) 15 (39%)
Mouthrinse use (before study)	No Yes	35 (95%) 2 (5%)	32 (89%) 4 (11%)	35 (92%) 3 (8%)
BOMP *P = 0.05 (Friedman test)*	Mean (SD) 95% CI Median (Range)	0.3 (0.2) 0.2–0.3 0.2 (0.1–0.7)	0.3 (0.2) 0.2–0.4 0.3 (0.1–0.9)	0.4 (0.2) 0.3–0.4 0.4 (0.1–0.9)
BOMP%	Mean (SD) 95% CI Median (Range)	19 (12) 15–23 17 (4–49)	21 (11) 18–21 19 (7–49)	26 (13) 22–30 26 (5–58)
PGE−2	Mean (SD) 95% CI Median (Range)	170 (147) 121–219 116 (13–585)	119 (71) 95–143 100 (25–337)	230 (258) 145–315 121 (2–1243)
IL1ß	Mean (SD) 95% CI Median (Range)	227 (212) 156–298 157 (0–1078)	225 (184) 163–288 205 (0–811)	235 (167) 180–290 219 (31–712)
MGI	Mean (SD) 95% CI Median (Range)	0.4 (0.3) 0.3–0.5 0.4 (0.02–1.4)	0.4 (0.2) 0.3–0.5 0.3 (0.1–0.9)	0.5 (0.3) 0.4–0.6 0.4 (0.1–1.4)
MGI%	Mean (SD) 95% CI Median (Range)	33 (19) 26–39 31 (2–85)	30 (14) 26–35 28 (7–58)	36 (19) 30–42 34 (6–88)
PI	Mean (SD) 95% CI Median (Range)	0.5 (0.3) 0.4–0.6 0.5 (0.1–0.9)	0.5 (0.2) 0.5–0.6 0.5 (0.1–1)	0.5 (0.2) 0.4–0.6 0.5 (0.1–0.9)
PI%	Mean (SD) 95% CI Median (Range)	44 (20) 38–51 45 (5–79)	48 (17) 43–54 49 (5–81)	45 (18) 39–51 48 (6–73)
RFP	Mean (SD) 95% CI Median (Range)	0.9 (1.2) 0.5–1.3 0.3 (0–4)	0.7 (0.8) 0.4–1 0.4 (0–4)	1 (1.3) 0.6–1.4 0.3 (0–6)
pH	Mean (SD) 95% CI Median (Range)	6.5 (0.4) 6.3–6.6 6.5 (5.5–7.1)	6.5 (0.5) 6.4–6.7 6.6 (5.2–7.4)	6.4 (0.5) 6.2–6.5 6.3 (5.3–7.4)
Diversity Index plaque sample	Mean (SD) 95% CI Median (Range)	3.7 (0.5) 3.5–3.9 3.8 (2.3–4.5)	3.7 (0.5) 3.5–3.9 3.8 (2–4.7)	3.6 (0.5) 3.4–3.8 3.7 (2.2–4.7)
Diversity Index saliva sample	Mean (SD) 95% CI Median (Range)	3.9 (0.3) 3.8–4 4 (3.1–4.4)	4 (0.3) 3.9–4.1 4 (3.3–4.4)	4 (0.3) 3.9–4.1 4 (3.1–4.5)
Diversity Index tongue sample	Mean (SD) 95% CI Median (Range)	3.7 (0.3) 3.6–3.8 3.7 (3–4.3)	3.7 (0.3) 3.6–3.8 3.8 (3.1–4.3)	3.7 (0.3) 3.6–3.8 3.8 (2.6–4.2)
Bacterial DNA in saliva sample (ng/μL)	Mean (SD) 95% CI Median (Range)	9.6 (7.0) 6.5–12.6 6.5 (2–26)	10.4 (9.4) 7.1–13.6 6.4 (0.7–37)	8.3 (5.8) 6.4–10.2 6.3 (1.8–25)
Bacterial DNA in tongue sample (ng/μL)	Mean (SD) 95% CI Median (Range)	5.8 (5.4) 3.5–8.1 3.9 (1.1–25)	5.2 (5.8) 3.2–7.2 3.7 (0.7–29)	4.5 (3.7) 3.3–5.8 3.4 (1–22)
Bacterial DNA in plaque sample (ng/μL)	Mean (SD) 95% CI Median (Range)	6.0 (3.6) 4.4–7.5 6.5 (1.2–13)	5.4 (3.9) 4.1–6.7 5.1 (0.01–16)	5.3 (3.7) 4.1–6.5 4.2 (0.03–19)
Fungal DNA in plaque sample (pg/μL)	Mean (SD) 95% CI Median (Range)	3.0 (13.5) −1.6–7.7 0.03 (0.0008–80)	28 (141.5) −20–77 0.04 (0.0008–835)	15.5 (68) −7–38 0.06 (0.0008–398)
Fungal load in plaque sample (%)	Mean (SD ) 95% CI Median (Range)	0.04 (0.14) −0.007–0.09 0.0005 (0.00002–0.8)	0.4 (1.6) −0.2–0.9 0.0007 (0.00002–9.4)	0.5 (2) −0.2–1.2 0.0007 (0.00001–9.6)
*L. paracasei* (gen. units/μL) in saliva	Mean (SD) 95% CI Median (Range)	7.2 (29) −2.7–17 0.4 (0–173)	6.2 (27) −2.8–15 0.08 (0–159)	19 (89) −11–49 0.3 (0–543)
*L. paracasei* (gen. units/μL) in tongue *P = 0.02 (Friedman test)*	Mean (SD) 95% CI Median (Range)	0.1 (0.5) −0.04–0.3 0 (0–2.7)	0.2 (0.8) −0.1–0.4 0 (0–5)	0.5 (1.5) −0.01–1.1 0 (0–9)
*L. paracasei* (gen. units/μL) in plaque	Mean (SD) 95% CI Median (Range)	0.1 (0.4) 0.002–0.3 0 (0–1.9)	0.04 (0.1) −0.004–0.09 0 (0–0.6)	0.2 (0.5) 0.01–0.3 0 (0–2.3)
*L. plantarum* (gen. units/μL) in saliva	Mean (SD) 95% CI Median (Range)	22.8 (98) −11–56 0 (0–552)	3 (13) −1.4–7.7 0 (0–78)	11 (64.5) −10–33 0 (0–393)
*L. plantarum* (gen. units/μL) in tongue	Mean (SD) 95% CI Median (Range)	0.4 (1.6) −0.15–0.9 0 (0–8)	0.4 (1.8) −0.2–1 0 (0–10)	0.08 (0.3) −0.025–0.19 0 (0–1.5)
*L. plantarum* (gen. units/μL) in plaque	Mean (SD) 95% CI Median (Range)	0.2 (1) −0.14–0.5 0 (0–5.6)	0.03 (0.2) −0.03–0.1 0 (0–1.2)	0.5 (2.8) −0.5–1.4 0 (0–17)

### Primary Study Outcome

The main study endpoint was the change in gingival bleeding on marginal probing (ΔBOMP index) between the end of the experimental gingivitis period (V5) and the baseline visit (V1). For group C, ΔBOMP V5V1 was not normally distributed (*p* = 0.026). After log10 transformation all three groups showed not normal data distribution (A: *p* = 0.002; B: *p* = 0.015; C: *p* = 0.008). Therefore, the differences among the groups were tested non-parametrically, on untransformed data. There was no difference found in the ΔBOMP between the visits 5 and 1 among the study groups (*p* = 0.905, Kruskal-Wallis test) (Table 2).

**Table 2:** Primary and secondary study outcomes in per-protocol (PP) population.

**Output**	**Group (N)**	**Mean (SD)**	**95% CI**	**Median (range)**
ΔBOMP V5V1	A(35)	0.67 (0.29)	0.57–0.77	0.70 (0.1–1.2)
	B(35)	0.67 (0.41)	0.53–0.80	0.67 (0.1–1.5)
	C(37)	0.65 (0.33)	0.54–0.76	0.62 (0.2–1.2)
ΔBOMP V3V1	A(35)	0.14 (0.16)	0.09–0.20	0.14 (−0.1–0.6)
	B(35)	0.13 (0.19)	0.06–0.20	0.12 (−0.2–0.8)
	C(37)	0.13 (0.18)	0.07–0.19	0.12 (−0.2–0.6)
ΔBOMP V6V1	A(35)	0.13 (0.15)	0.08–0.18	0.12 (−0.1–0.5)
	B(35)	0.17 (0.23)	0.09–0.25	0.10 (−0.2–0.8)
	C(37)	0.15 (0.20)	0.08–0.22	0.08 (−0.1–0.9)
ΔBOMP% V5V1	A(35)	38 (13)	32–43	39 (2–66)
	B(35)	38 (21)	30–45	41 (1–78)
	C(37)	36 (18)	30–42	37 (6–66)
ΔBOMP% V3V1	A(35)	9 (11)	6–13	11 (−8–35)
	B(35)	9 (12)	5–13	10 (−11–39)
	C(37)	8 (11)	4–11	7 (−13–29)
ΔBOMP% V6V1	A(35)	7 (10)	4–10	7 (−7–32)
	B(35)	10 (13)	5–14	6 (−11–43)
	C(37)	7 (11)	3–11	8 (−12–42)
ΔPGE2 V3V1	A(35)	−27 (79)	−54–0.4	−11 (−298–117)
	B(35)	64 (344)	−54–182	−2 (−190–1976)
	C(37)	37 (424)	−105–178	7 (−1150–2148)
ΔPGE2 V4V1	A(35)	28 (344)	−90–146	−14 (−291–1948)
	B(35)	40 (96)	6.7–73	28 (−111–351)
	C(37)	−31 (268)	−120–59	−4 (−1081–701)
ΔPGE2 V5V1	A(35)	−1.3 (114)	−40–38	−6 (−211–473)
	B(35)	42 (121)	0.1–83	8.8 (−281–330)
	C(37)	−3.2 (246)	−85–79	27 (−1132–613)
ΔPGE2 V6V1	A(35)	−23 (197)	−91–45	−20 (−367–952)
	B(35)	32 (123)	−10–74	6 (−181–470)
	C(37)	−19 (252)	−103–65	5 (−1008–727)
ΔIL1ß V3V1	A(35)	−15 (176)	−75–46	−24 (−684–433)
	B(35)	62 (280)	−34–158	50 (−602–1209)
	C(37)	46 (169)	−10–103	27 (−244–448)
ΔIL1ß V4V1	A(35)	−0.4 (147)	−51–50	14 (−407–471)
	B(35)	106 (220)	31–182	70 (−184–1011)
	C(37)	58 (224)	−17–132	38 (−377–744)
ΔIL1ß V5V1	A(35)	20 (178)	−41–81	0 (−704–336)
	B(35)	92 (164)	35–148	55 (−178–512)
	C(37)	78 (231)	1.5–155	59 (−486–719)
ΔIL1ß V6V1	A(35)	−3.7 (161)	−59–52	−11 (−438–479)
	B(35)	27 (129)	−17–71	38 (−459–282)
	C(37)	51 (253)	−34–135	22 (−538–818)–
ΔMGI V3V1	A(35)	0.01 (0.2)	−0.06–0.09	0.01 (−0.5–0.5)
	B(35)	0.1 (0.3)	0.004–0.2	0.01 (−0.03–0.9)
	C(37)	0.1 (0.3)	−0.03–0.2	0.1 (−0.4–0.7)
ΔMGI V5V1	A(35)	0.8 (0.2)	0.7–0.9	0.8 (0.3–1.3)
	B(35)	0.8 (0.3)	0.7–0.9	0.7 (0.4–1.5)
	C(37)	0.8 (0.3)	0.7–0.9	0.8 (0.3–1.4)
ΔMGI V6V1 *P = 0.024 (Friedman Test)*	A(35)	0.1 (0.3)	−0.01–0.2	0.1 (−0.8–0.6)
	B(35)	0.3 (0.3)	0.2–0.4	0.3 (−0.5–0.9)
	C(37)	0.1 (0.2)	0.1–0.2	0.1 (−0.4–0.6)
ΔMGI% V3V1	A(35)	2.4 (16)	−3–8	3.6 (−31–40)
	B(35)	7 (20)	0.1–14	1.2 (−25–54)
	C(37)	6 (20)	−0.6–13	8 (−31–58)
ΔMGI% V5V1	A(35)	55 (16)	49–60	55 (14–82)
	B(35)	55 (14)	51–60	56 (27–83)
	C(37)	56 (18)	50–62	56 (12–86)
ΔMGI% V6V1	A(35)	11 (23)	3.5–19	10 (−32–63)
	B(35)	23 (23)	15–31	24 (−39–70)
	C(37)	16 (18)	9.5–22	18 (−25–51)
ΔPI V3V1	A(34)	0.03 (0.2)	−0.05–0.1	0.02 (−0.6–0.5)
	B(35)	0.04 (0.2)	−0.02–0.1	0.02 (−0.4–0.5)
	C(35)	0.08 (0.2)	0.02–0.2	0.08 (−0.4–0.6)
ΔPI V5V1	A(34)	1.1 (0.2)	1–1.2	1 (0.5–1.6)
	B(35)	1.1 (0.3)	1–1.2	1 (0.5–1.6)
	C(35)	1 (0.3)	0.9–1.1	1 (0.3–2)
ΔPI V6V1	A(34)	0.2 (0.3)	0.1–0.2	0.1 (−0.5–0.6)
	B(35)	0.1 (0.2)	0.1–0.2	0.2 (−0.4–0.6)
	C(35)	0.2 (0.3)	0.1–0.3	0.2 (−0.5–1)
ΔPI% V3V1	A(34)	5.5 (19)	−1.1–12	6.6 (−45–53)
	B(35)	5.9 (16)	0.5–11	6 (−25–50)
	C(35)	10 (16)	4–15	11 (−30–39)
ΔPI% V5V1	A(34)	49 (20)	42–55	48 (10–93)
	B(35)	47 (18)	41–53	45 (19–93)
	C(35)	48 (16)	43–54	48 (18–81)
ΔPI% V6V1	A(34)	13 (21)	6–20	14 (−43–52)
	B(35)	11 (19)	5–18	13 (−32–54)
	C(35)	19 (24)	11–27	19 (−42–71)
ΔRFP% V2V1	A(34)	0.03 (0.7)	−0.2–0.3	0.03 (−2–3)
	B(34)	0.2 (0.6)	−0.03–0.4	0.03 (−0.7–2)
	C(33)	0.05 (1.5)	−0.5–0.6	−0.004 (−4–5)
ΔRFP% V3V1	A(34)	−0.3 (0.4)	−0.4–−0.1	−0.1 (−1–0.3)
	B(34)	−0.1 (0.7)	−0.4–0.1	−0.01 (−3–1)
	C(33)	0.1 (0.7)	−0.2–0.3	−0.02 (−1–3)
ΔRFP% V4V1	A(34)	1.6 (1.4)	1.1–2.1	1.4 (−0.1–5)
	B(34)	1.8 (1.9)	1.1–2.4	1.1 (−0.5–8)
	C(33)	1.8 (2.2)	1.1–2.6	0.7 (−0.4–8)
ΔRFP% V5V1	A(34)	3.2 (3.8)	1.9–4.5	2 (0.1–20)
	B(34)	3 (3.6)	1.9–4.4	1.8 (−0.2–17)
	C(33)	3.5 (4.6)	1.9–5	2.3 (−0.5–22)
ΔRFP% V6V1	A(34)	−0.1 (1.1)	−0.4–0.3	0.001 (−3–3)
	B(34)	0.1 (1.1)	−0.3–0.5	0.005 (−3–3)
	C(33)	0.3 (1.8)	−0.4–0.9	−0.01 (−2–8)
ΔpH V3V1	A(34)	−0.4 (0.6)	−0.6– −0.2	−0.4 (−1.3–1.3)
	B(33)	−0.1 (0.6)	−0.3–0.1	−0.1 (−1–1)
	C(35)	−0.2 (0.7)	−0.4–0.1	−0.3 (−1.7–1.2)
ΔDiv plaque V2V1	A(35)	0.2 (0.3)	0.03–0.3	0.1 (−0.6–0.9)
	B(34)	0.2 (0.5)	0.1–0.4	0.2 (−0.7–1.5)
	C(36)	0.1 (0.5)	−0.1–0.2	0.2 (−0.7–1.5)
ΔDiv plaque V3V1	A(35)	0.1 (0.4)	−0.04–0.3	0.2 (−1.2–1)
	B(34)	0.3 (0.6)	0.1–0.4	0.3 (−1–1.4)
	C(37)	0.2 (0.4)	0.1–0.4	0.2 (−0.8–1.1)
ΔDiv plaque V4V1	A(34)	0.8 (0.5)	0.6–0.9	0.7 (−0.2–1.5)
	B(34)	0.7 (0.6)	0.5–0.9	0.6 (−0.1–3)
	C(37)	0.7 (0.7)	0.5–1	0.8 (−1.3–2)
ΔDiv plaque V5V1	A(35)	0.7 (0.5)	0.5–0.9	0.6 (−0.5–1.8)
	B(34)	0.8 (0.6)	0.5–1	0.7 (−0.2–2.4)
	C(36)	0.8 (0.6)	0.6–1	0.8 (−1.4–2)
ΔDiv plaque V6V1	A(35)	0.1 (0.5)	−0.1–0.3	0.1 (−1–1)
	B(34)	0.1 (0.6)	−0.1–0.3	0.1 (−1.5–1.3)
	C(35)	0.1 (0.6)	−0.1–0.3	0.2 (−1.5–1)
ΔDiv saliva V2V1	A(35)	0.1 (0.3)	−0.03–0.2	0.1 (−0.8–0.7)
	B(34)	0.03 (0.3)	−0.1–0.1	0.03 (−0.4–0.8)
	C(37)	0.1 (0.2)	−0.02–0.1	0.1 (−0.6–0.7)
ΔDiv saliva V3V1	A(34)	0.1 (0.2)	0.01–0.2	0.1 (−0.4–0.4)
	B(35)	0.1 (0.3)	−0.1–0.1	−0.01 (−0.8–0.6)
	C(36)	0.04 (0.3)	−0.1–0.1	0.1 (−0.4–0.8)
ΔDiv saliva V4V1	A(35)	0.3 (0.3)	0.2–0.4	0.3 (−0.3–1.1)
	B(35)	0.2 (0.3)	0.1–0.3	0.2 (−0.4–0.9)
	C(37)	0.2 (0.3)	0.1–0.3	0.2 (−0.5–1)
ΔDiv Saliva V5V1	A(35)	0.3 (0.3)	0.2–0.4	0.3 (−0.3–0.9)
	B(35)	0.3 (0.3)	0.2–0.4	0.3 (−0.6–0.8)
	C(37)	0.3 (0.2)	0.2–0.4	0.3 (−0.2–0.8)
ΔDiv saliva V6V1	A(35)	0.1 (0.3)	−0.1–0.2	0.03 (−0.6–1)
	B(35)	0.1 (0.2)	0.02–0.1	0.1 (−0.4–0.5)
	C(37)	−0.02 (0.3)	−0.1–0.1	−0.03 (−0.5–0.7)
ΔDiv tongue V2V1	A(34)	−0.03 (0.3)	−0.1–0.1	−0.1 (−0.7–0.6)
	B(33)	−0.1 (0.3)	−0.2–0.1	−0.1 (−0.8–0.5)
	C(36)	−0.1 (0.3)	−0.2–0.02	−0.1 (−0.6–0.4)
ΔDiv tongue V3V1	A(34)	−0.003 (0.4)	−0.1–0.1	−0.02 (−1–0.7)
	B(35)	−0.03 (0.3)	−0.1–0.1	0.03 (−0.7–0.6)
	C(36)	0.01 (0.3)	−0.1–0.1	0.02 (−0.8–0.6)
ΔDiv tongue V4V1	A(34)	0.05 (0.3)	−0.1–0.1	0.04 (−0.6–0.5)
	B(35)	−0.02 (0.3)	−0.1–0.1	−0.03 (−0.5–0.5)
	C(37)	0.1 (0.3)	−0.1–0.2	0.02 (−1–0.8)
ΔDiv tongue V5V1	A(32)	0.1 (0.4)	−0.04–0.2	0.04 (−0.6–0.8)
	B(35)	0.05 (0.3)	−0.05–0.1	−0.01 (−0.6–0.5)
	C(36)	0.1 (0.4)	−0.03–0.2	0.1 (−0.6–1.2)
ΔDiv tongue V6V1	A(33)	0.03 (0.3)	−0.1–0.1	0.001 (−0.5–1)
	B(35)	0.05 (0.3)	−0.05–0.1	0.1 (−0.6–0.5)
	C(37)	−0.02 (0.3)	−0.1–0.1	0.03 (−0.7–0.5)
ΔBacterial DNA saliva V2V1	A(35)	−0.7 (4.8)	−2.3–1	−1.1 (−11–12)
	B(35)	−2 (6.2)	−4–0.01	−0.9 (−21–7)
	C(37)	1 (5.2)	−0.8–2.7	0.1 (−9–24)
ΔBacterial DNA saliva V3V1	A(35)	0.01 (5.7)	−1.9–2	−0.8 (−9–20)
	B(35)	0.0002 (6)	−2–2	0.2 (−15–16)
	C(37)	2.5 (8.6)	−0.4–5.4	0.8 (−9–35)
ΔBacterial DNA saliva V4V1	A(35)	−0.7 (5.9)	−2.8–1.3	−1.2 (−12–16)
	B(35)	0.5 (5.6)	−1.4–2.4	0.5 (−14–12)
	C(37)	1.6 (8)	−1.1–4.3	1.8 (−16–27)
ΔBacterial DNA saliva V5V1	A(35)	0.5 (4.4)	−1–2	−0.3 (−9–11)
	B(35)	−0.7 (6)	−2.8–1.3	−0.4 (−14–9)
	C(37)	0.8 (7.5)	−1.7–3.3	−0.9 (−14–34)
ΔBacterial DNA saliva V6V1	A(35)	0.5 (6.3)	−1.7–2.6	−0.4 (−12–21)
	B(35)	−1.5 (6)	−3.6–0.6	−0.2 (−16–8)
	C(37)	3.1 (9.4)	−0.02–6.3	1.4 (−17–27)
ΔBacterial DNA plaque V2V1	A(35)	1.6 (3.2)	0.5–2.7	0.4 (−3–8.5)
	B(35)	1.1 (4.4)	−0.5–2.6	1.1 (−11–11)
	C(37)	0.6 (3.9)	−0.7–2	1.1 (−8.5–9)
ΔBacterial DNA plaque V3V1	A(35)	0.9 (3.9)	−0.5–2.2	−0.25 (−4–11)
	B(35)	1 (4.6)	−0.6–2.6	0.9 (−11–14)
	C(37)	0.3 (3.9)	−1–1.6	0.1 (−12–7)
ΔBacterial DNA plaque V4V1	A(35)	3.5 (6.1)	1.4–5.6	1.6 (−8–18)
	B(35)	5 (8.7)	2–8	3 (−7.6–36)
	C(37)	3.1 (5.7)	1.2–5	2.4 (−10–15)
ΔBacterial DNA plaque V5V1	A(35)	1.6 (6.1)	−0.5–3.7	0.4 (−9–15)
	B(35)	3.8 (7.6)	1.2–6.4	1.4 (−10–20)
	C(37)	3.2 (7.2)	0.8–5.6	1.4 (−9–22)
ΔBacterial DNA plaque V6V1	A(35)	0.1 (4)	−1.3–1.5	−0.5 (−9–9)
	B(35)	0.5 (3.6)	−0.7–1.8	0.2 (−7–11)
	C(37)	−0.4 (4.1)	−1.8–1	−0.4 (−12–10)
ΔBacterial DNA tongue V2V1	A(35)	−2 (2.6)	−2.9– −1.1	−1.6 (−10–3.8)
	B(35)	−1.8 (4.7)	−3.4– −0.1	−1.2 (−20–6)
	C(37)	−1.2 (2)	−1.9– −0.5	−1.3 (−8–3)
ΔBacterial DNA tongue V3V1	A(35)	−2.4 (2.4)	−3.2– −1.5	−1.7 (−8–1)
	B(35)	−1.4 (4.4)	−2.9–0.1	−1.2 (−15–11)
	C(37)	−0.9 (3.9)	−2.2–0.4	−1 (−6–18)
ΔBacterial DNA tongue V4V1 *P = 0.036 (Friedman Test)*	A(35)	−2.6 (2.8)	−3.5– −1.6	−1.8 (−12–0.5)
	B(35)	−1.3 (3.7)	−2.6– −0.004	−0.8 (−14–5)
	C(37)	−1.5 (2.8)	−2.4– −0.6	−0.9 (−11–3)
ΔBacterial DNA tongue V5V1	A(35)	−2.8 (2.8)	−3.7– −1.8	−2.3 (−9–1.9)
	B(35)	−1.5 (3.7)	−2.7– −0.2	−1.1 (−13–8)
	C(37)	−2.2 (2.9)	−3.2– −1.3	−1.8 (−14–3)
ΔBacterial DNA tongue V6V1	A(35)	−1.9 (3.2)	−3– −0.8	−1.7 (−9–7)
	B(35)	−1.6 (4.2)	−3– −0.14	−0.5 (−16–3)
	C(37)	−1.8 (3.9)	−3.1– −0.5	−1.7 (−17–7)
ΔFungal DNA plaque V2V1 *P = 0.04 (Friedman test)*	A(35)	9.7 (30.5)	−0.8–20.2	0.03 (−6–142)
	B(35)	−3.7 (77.7)	−30–23	0 (−419–131)
	C(37)	14.5 (149)	−35–64	0 (−307–840)
ΔFungal DNA plaque V3V1 *P = 0.04 (Friedman Test)*	A(35)	7.3 (31)	−3.2–18	0.02 (−6–176)
	B(35)	16.6 (44)	1.5–32	0.03 (−0.8–207)
	C(37)	−6 (79)	−32–20	0 (−377–256)
ΔFungal DNA plaque V4V1	A(35)	9.4 (53)	−8.9–28	0.04 (−70–300)
	B(35)	−14 (132)	−59–31	0 (−750–182)
	C(37)	22 (108)	−14–58	0.006 (−91–538)
Δfungal DNA plaque V5V1	A(35)	−1.1 (14)	−6–3.8	0 (−78–27)
	B(35)	35 (337)	−80–151	0 (−746–1823)
	C(37)	−13 (56)	−31–5.7	0 (−320–8.7)
Δfungal DNA plaque V6V1	A(35)	6 (35)	−6–18)	0.0007 (−19–205)
	B(35)	44 (222)	−32–120	0 (−105–1256)
	C(37)	−11 (59)	−31–8.6	0.009 (−332–70)
Δfungal load plaque V2V1	A(35)	0.2 (0.5)	−0.03–0.3	0.0005 (−0.8–2.9)
	B(35)	0.07 (0.5)	−0.1–0.2	0 (−1.9–1.8)
	C(37)	0.7 (3.8)	−0.6–2	0 (−6.6–18)
Δfungal load plaque V3V1 *P = 0.04 (Friedman test)*	A(35)	0.4 (1.4)	−0.12–0.9	0.0002 (−0.8–6)
	B(35)	0.3 (0.8)	−0.2–0.5	0.0009 (−0.1–4)
	C(37)	−0.4 (2)	−1–0.35)	0 (−9–4)
ΔFungal load plaque V4V1	A(35)	0.2 (0.9)	−0.1–0.5	0.0002 (−0.7–5)
	B(35)	−0.2 (1)	−0.5–0.2	0 (−6–0.5)
	C(37)	0.2 (1.6)	−0.4–0.7	0 (−4.6–8)
ΔFungal load plaque V5V1	A(35)	0.0007 (0.2)	−0.05–0.05	0 (−0.7–0.4)
	B(35)	0.01 (2)	−0.6–0.7	−0.0001 (−7–8)
	C(37)	−0.4 (1.8)	−1–0.2	−0.0001 (−8–0.04)
ΔFungal load plaque V6V1	A(35)	0.2 (1)	−0.2–0.5	0 (−0.3–6)
	B(35)	1.4 (8)	−1.4–4.2	0 (−2–48)
	C(37)	0.6 (4.6)	−1–2	0.0002 (−7–26)

### Secondary and Other Study Outcomes

Among the secondary study outcomes, a significant difference was found among the study groups in the change of Modified Gingival Index (MGI) between visits 1 and 6 (ΔMGI V6V1) (*p* = 0.024, Kruskal-Wallis test) (Table 2). Group B showed a significantly larger change in MGI than group A (*p* = 0.0167, Mann-Whitney test, FDR corrected). In other words, gingival inflammation of the subjects in group B had recovered less from the experimental gingivitis after the recovery phase (visit 6) than in the subjects in group A. No significant differences were found in group C.

Significant difference among the groups was also observed in the change of bacterial DNA concentration in the tongue samples between visits 1 and 4 (Δ Bacterial DNA tongue V4V1) (*p* = 0.036, Kruskal-Wallis test) (Table 2). Group A showed a significantly larger reduction in bacterial DNA concentration than group B (*p* = 0.0167, Mann-Whitney test, FDR corrected). A similar trend was observed between visits 1 and 5 (Δ Bacterial DNA tongue V5V1), just above the significance threshold (*p* = 0.059). This means that individuals in group A accumulated significantly fewer bacteria on their tongues during the experimental gingivitis period.

Fungal DNA concentration was measured in dental plaque samples only. Here the significant increase in fungal DNA concentration was observed between V1 and V2 (Δ V2V1) in group A samples in comparison to group C (*p* = 0.04 Kruskal-Wallis test, *p* = 0.0167 Mann-Whitney test, FDR corrected) and between V1 and V3 in group B compared to group C samples (*p* = 0.042 Kruskal-Wallis test, *p* = 0.0167 Mann-Whitney test, FDR corrected). There was a significant increase in fungal load (% fungal DNA over bacterial DNA) between V1 and V3 (*p* = 0.04, Kruskal-Wallis test) in group A and group C compared to group B samples, though this significance was lost after FDR correction for multiple comparisons (Table 2).

To assess if the treatments induced significant differences among the three groups in the microbiome of salivary, plaque, and tongue samples, PERMANOVA on microbial profile data was performed. No significant differences among the groups at any of the sample types and any of the study visits were observed, although a nearly significant *p*-value (*p* = 0.07, *F* = 1.28) was obtained among the three groups of saliva samples, collected at V4 (Supplementary Figure 2).

### Ancillary Analyses

**Figure 3:**
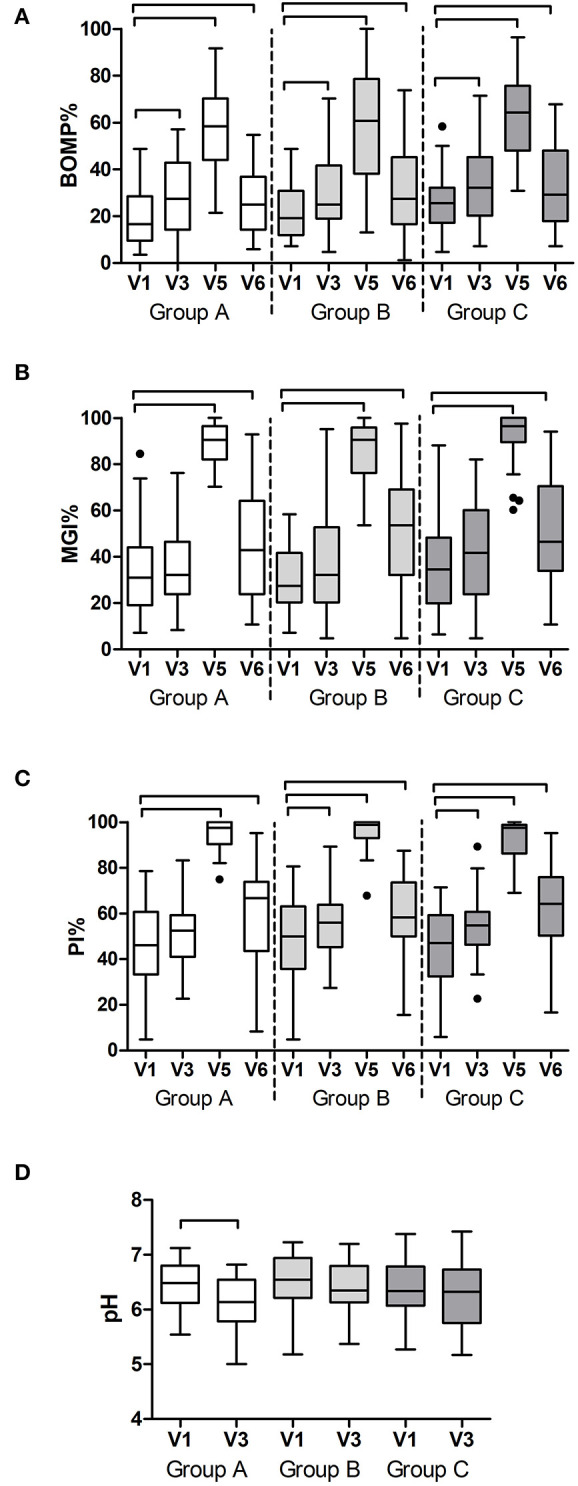
Clinical findings per study group and visit: **(A)** prevalence of bleeding on marginal probing (BOMP%); **(B)** prevalence of gingival inflammation (MGI%); **(C)** prevalence of dental plaque (PI%) as a percentage of all sites at the baseline (V1), end of the wash-in period (V3), end of the experimental gingivitis period (V5) and end of the wash-out period (V6); and **(D)** pH of the dental plaque at the baseline visit (V1) and at the end of the wash-in period (V3). The connectors connect the visits that differed significantly (*P* < 0.05, Wilcoxon Signed Ranks test) from the baseline visit.

**Figure 4:**
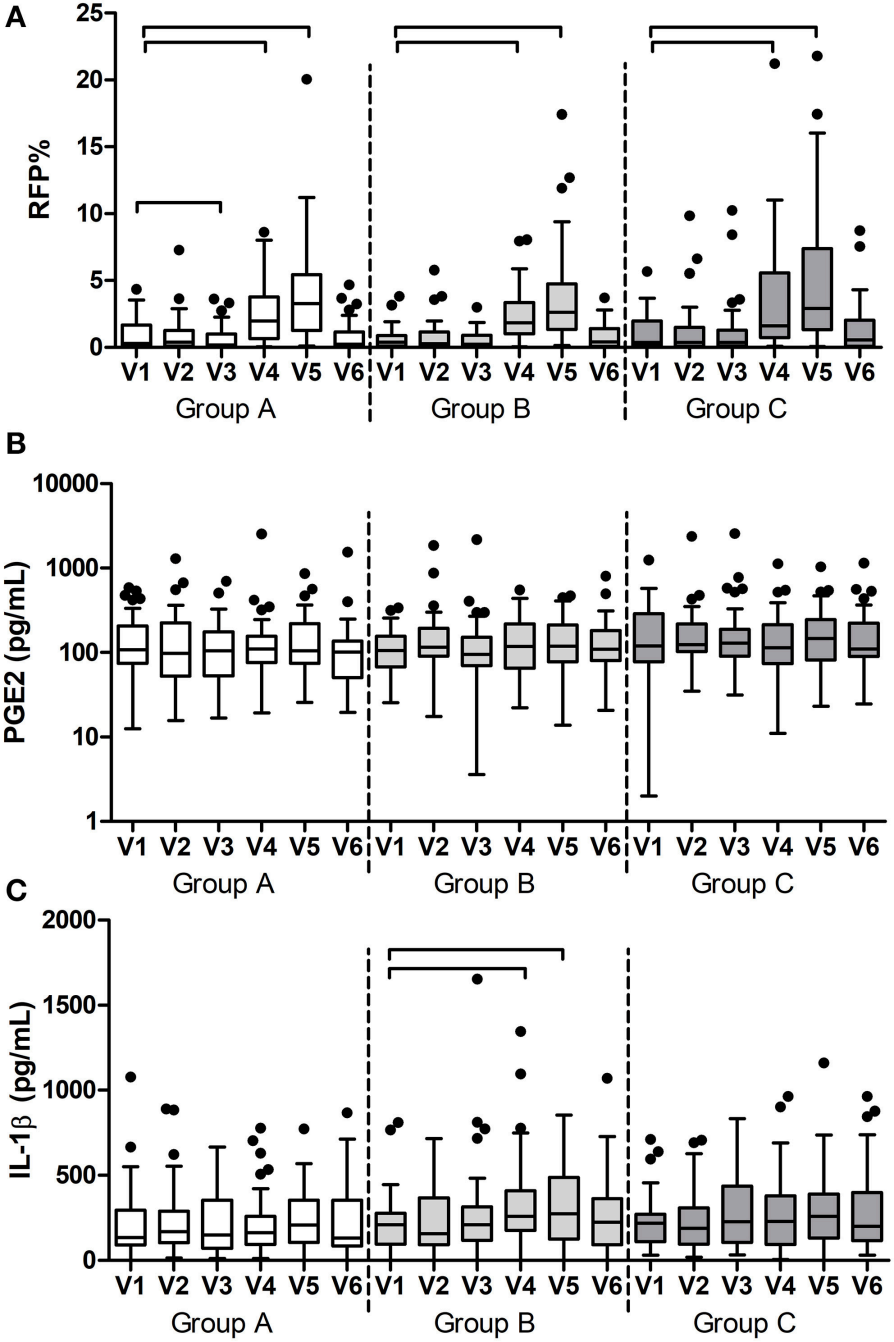
Results of the additional measurements per group and visit: **(A)** prevalence of red fluorescing plaque (RFP%); **(B)** concentration of prostaglandin E2 (PGE2) and **(C)** concentration of interleukin-1ß (IL-1ß) in saliva. The connectors connect the visits that differed significantly (*P* < 0.05, Wilcoxon Signed Ranks test) from the baseline visit.

Additional to the outcomes reported above, all variables were assessed in time within each group. As expected, gingival bleeding (BOMP, %BOMP), gingival inflammation (MGI, %MGI), plaque index (PI, %PI) (Figure 3), Supplementary Figure 3), and red fluorescing plaque (%RFP) (Figure 4A) all significantly increased during the experimental gingivitis phase and decreased in the recovery phase, irrespective of the study group. Among the parameters above, only RPF% returned to the baseline values after the recovery phase. Plaque prevalence (PI%) increased significantly during the wash-in period in groups B (*p* = 0.039, Wilcoxon Signed Ranks Test) and C (*p* = 0.02), while this did not occur in group A. Additionally, in group A, %RFP significantly decreased (*p* = 0.006, Wilcoxon Signed Ranks Test) in the wash-in phase between the baseline (V1) and the V3 2 weeks later. This was not observed in the other two groups.

At two study visits, which were V1 and V3, plaque pH was measured *ex vivo*, in order to assess the effects of the wash-in phase on the resting plaque pH. In group A, there was a significant decrease in plaque pH after the wash-in phase (*p* = 0.001, Paired Samples *t*-test), while no significant changes were measured in groups B and C (Figure 3D).

Among the two immunological parameters, namely PGE2 and IL1-ß, that were measured in saliva samples (Figures 4B,C), PGE2 did not change at any time in any of the groups, while IL1-ß significantly increased during the experimental gingivitis phase only in the group B (*p* < 0.0001, Friedman Test). A *post-hoc* analysis revealed that IL1-ß increased in saliva of the individuals from group B already after the first week of experimental gingivitis (V4) (*p* = 0.003, Wilcoxon Signed Ranks Test) and remained significantly higher after the second week (V5) (*p* = 0.038).

**Figure 5:**
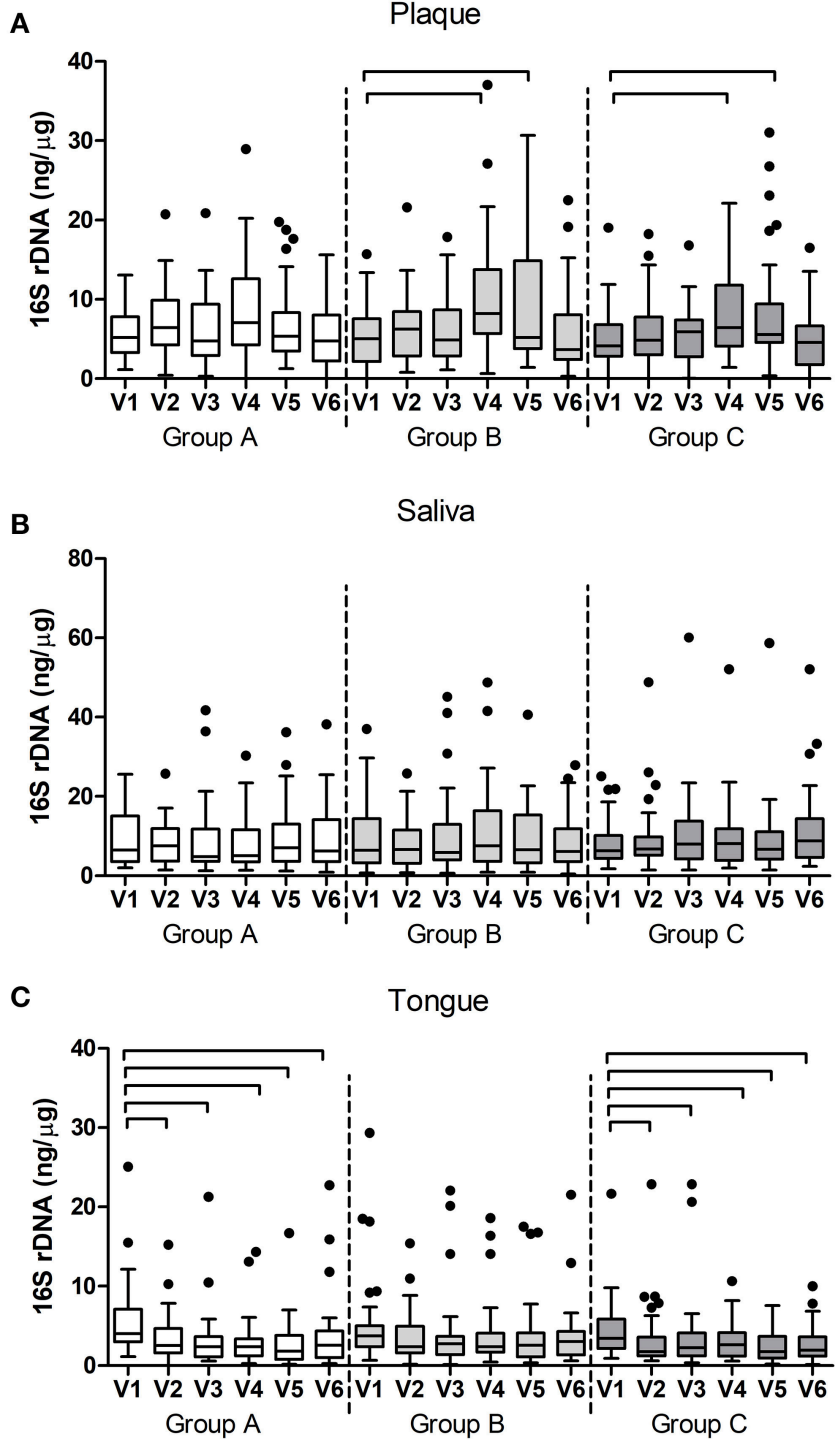
Bacterial DNA (16S rDNA) per group and per visit in **(A)** dental plaque, **(B)** salivary and **(C)** tongue samples. The connectors connect the visits that differed significantly (*P* < 0.05, Wilcoxon Signed Ranks test) from the baseline visit.

Bacterial DNA concentration was measured using qPCR targeting the 16S rRNA gene. In plaque, bacterial DNA concentration significantly changed in the time between the visits in group B (*p* < 0.0001, Friedman Test) and in group C (*p* < 0.0001), while no significant change was observed in group A (*p* = 0.057). *Post-hoc* analyses revealed that bacterial DNA significantly increased at V4 and V5 in groups B (*p* = 0.001 and *p* 0.009) and C (*p* = 0.002 and *p* = 0.029) during the experimental gingivitis period (Figure 5A). In saliva, no significant changes in bacterial DNA in time were observed (*p* > 0.05, Friedman test, Figure 5B), while in tongue samples, groups A and C changed significantly (*p* < 0.0001). In these samples, bacterial DNA significantly decreased from baseline visit (V1) to the later time points including the wash-out visit V6, with this decrease being the most pronounced in group A (*p* < 0.0001, Wilcoxon Signed Ranks Test) (Figure 5C).

**Figure 6:**
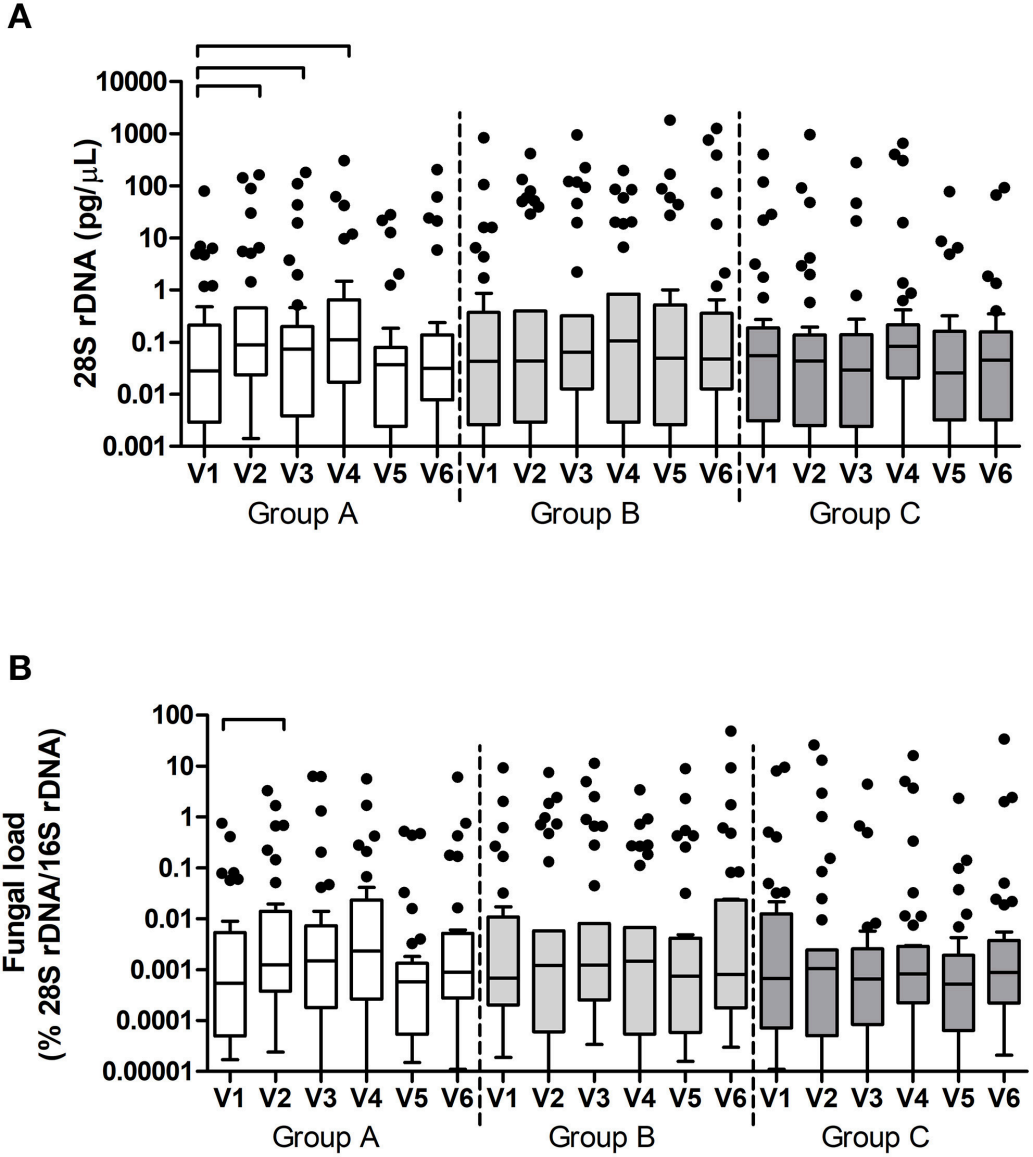
Fungal DNA (28S rDNA) **(A)** and Fungal load (fungal DNA relative to bacterial DNA) **(B)** per group and per visit in dental plaque samples. The connectors connect the visits that differed significantly (*P* < 0.05, Wilcoxon Signed Ranks test) from the baseline visit.

Fungal DNA concentrations were measured using qPCR targeting the fungal 28S rRNA gene in plaque samples. In time, only samples in group A showed a significant change in fungal DNA (*p* = 0.001, Friedman test). *Post-hoc* analyses revealed that this difference was due to a significant increase in fungal DNA in plaque samples between V1 and V2 (p = 0.002, Wilcoxon Signed Ranks Test), V1, and V3 (*p* = 0.041), and V1 and V4 (*p* = 0.033) (Figure 6). Fungal load also changed significantly in group A (*p* = 0.003) and was due to a significant increase in fungal load between V1 and V2 (*p* = 0.002).

To assess the presence of the test product in the samples, we performed targeted qPCR of the two lactobacilli species – *Lactobacillus plantarum* and *Lactobacillus paracasei* in the samples collected at V1, V3, V5, and V6 and we assessed the sequencing reads classified to genus *Lactobacillus* in all study visits. For the latter, across all sample types, there were 27 zOTUs classified as genus *Lactobacillus* (Supplementary Table 7). The majority (73%) of the lactobacilli reads originated from saliva samples, followed by the tongue (22%) and plaque (5%). The distribution of the top 8 most abundant lactobacilli zOTUs was similar in saliva and tongue samples – zOTU#143 and zOTU#262 together accounted for about 90% of the lactobacilli reads. In plaque, 44% of the lactobacilli reads belonged to the sum of the minor zOTUs and 32% were assigned to zOTU#455. The inter-group comparisons showed that saliva and tongue samples differed in zOTU #143 (group A) and zOTU #262 (group C) (Supplementary Figure 4), as well as two smaller zOTUs #1419 and #571 (in group C) at visits 2 through 5. Samples from group B had a negligible number of samples positive with these three zOTUs.

**Figure 7:**
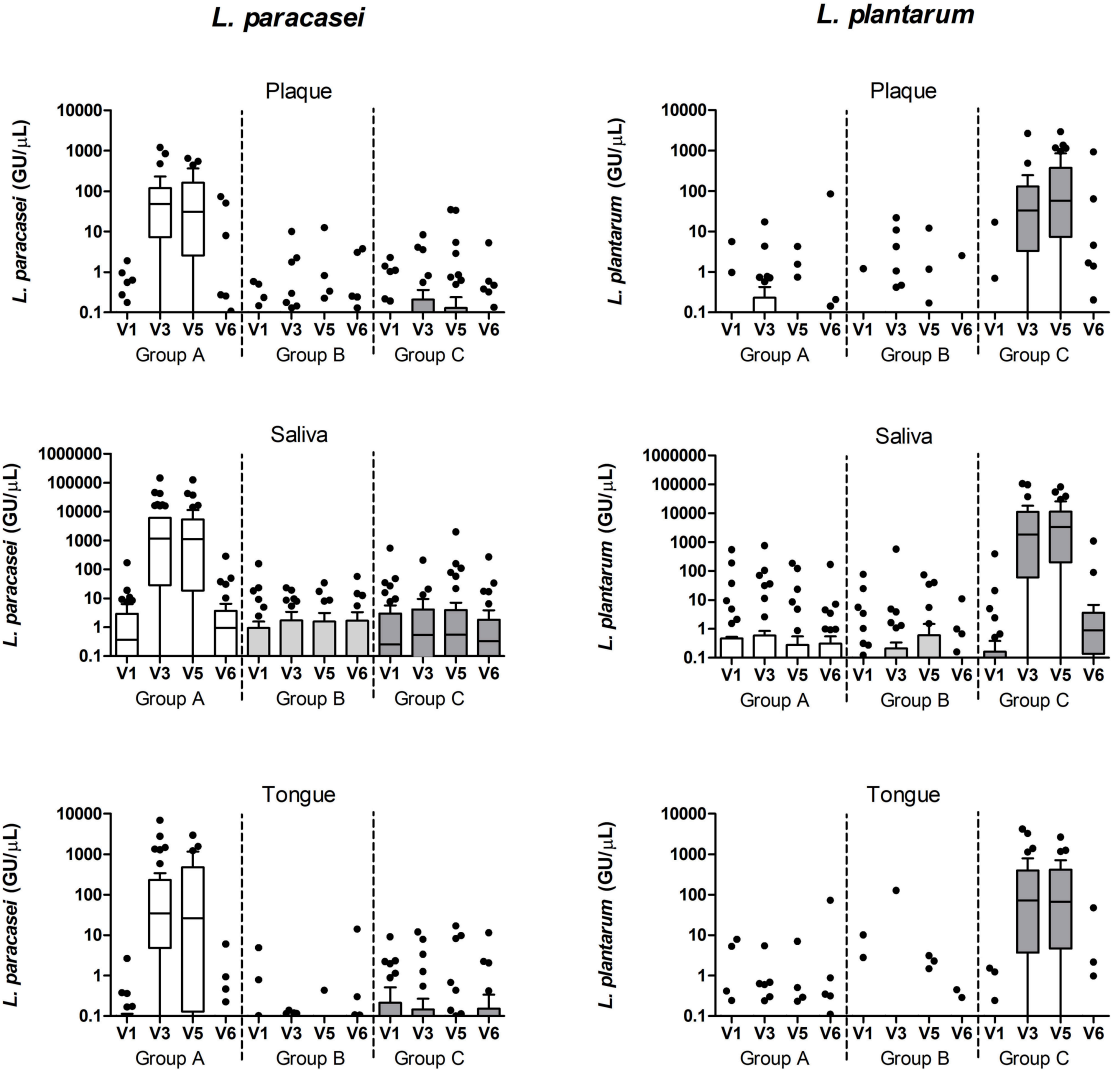
*L. paracasei* and *L. plantarum* qPCR counts (genomic units/μL sample) in plaque, saliva, and tongue samples per study group and visit.

The above findings were confirmed with targeted qPCR of the two lactobacilli species – *L. plantarum* and *L. paracasei* (Figure 7). Baseline samples of tongue and plaque samples contained between 0 and 17 (median 0) genomic units of the lactobacilli, while in baseline saliva samples between 0 and 552 (median 0–0.4) genomic units of the targeted lactobacilli were found (Table 1). After the wash-in period (V3) and also after the experimental gingivitis period (V5), the groups significantly differed from each other in all three sample types by the counts of both lactobacilli species (*p* < 0.0001). *L. paracasei* was significantly higher in group A than in group B or in group C (*p* < 0.0001), while *L. plantarum* was found significantly higher in group C than in group A or B (*p* < 0.0001). For both lactobacilli species, significantly higher counts were found in saliva than in plaque or tongue samples at all timepoints irrespective of the group (*p* < 0.001 for *L. paracasei*, and *p* < 0.01 for *L. plantarum*), while no difference in lactobacilli counts was observed between the plaque and tongue samples. At the end of the study, after the 2-week wash-out period without the use of the lozenges, the differences among the groups were lost except for *L. plantarum* in saliva (*p* < 0.0001), where samples from the group C still had statistically significantly higher counts than the other two groups (A: *p* = 0.0333, B: *p* = 0.0167, Mann-Whitney test, FDR corrected).

**Figure 8:**
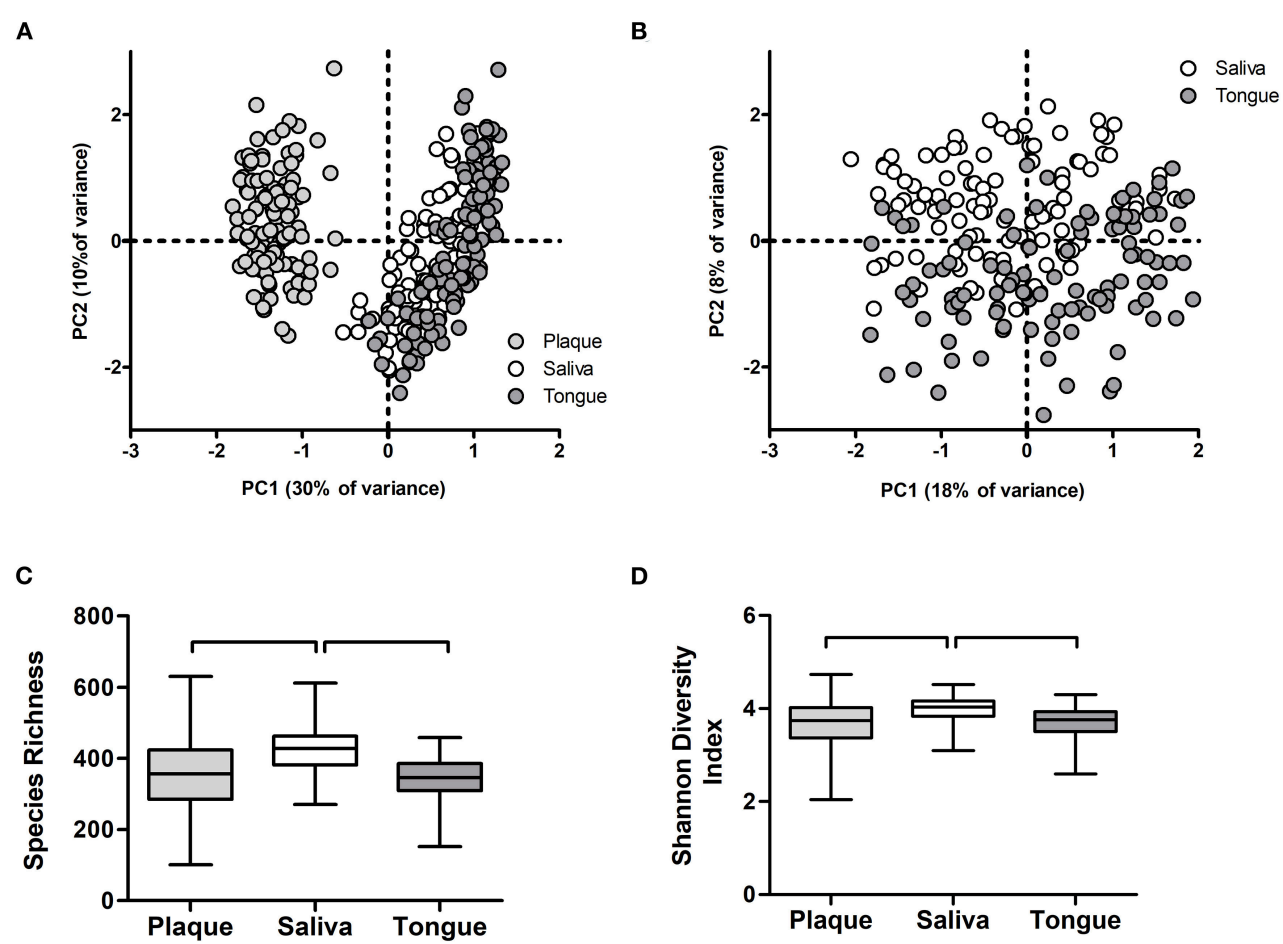
Microbiome output of the baseline samples: **(A)** Principal Component Analysis (PCA) of plaque, salivary, and tongue samples; **(B)** PCA of salivary and tongue samples; **(C)** species richness (zOTUs/sample) and **(D)** Shannon Diversity Index per sample type. Connectors indicate statistically significant differences (*p* < 0.05, Wilcoxon Signed Ranks test).

**Figure 9:**
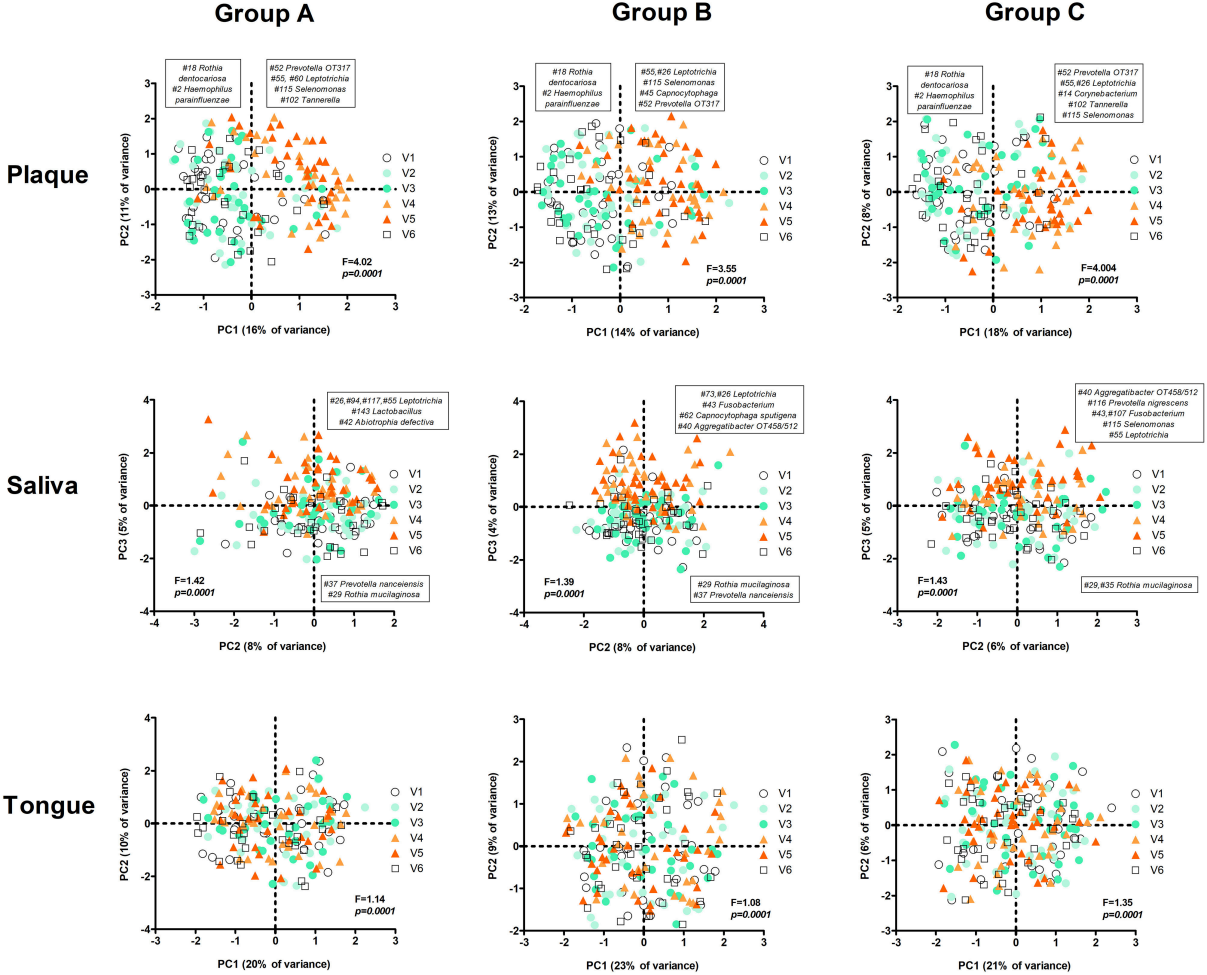
PCA plots per group and sample type by visit. The text boxes indicate the zOTUs that were the main loadings (arbitrary threshold 0.1/−0.1) of the principal components that were associated with experimental gingivitis (V4 and V5): PC1 (left – negative loadings, right – positive loadings) in plaque and PC3 in saliva samples (upper half of het plot – positive loadings, the lower part of the plot – negative loadings). No specific component associated with V4 and V5 was identified for the tongue samples. F and *p*-value – restricted PERMANOVA.

Next, we assessed the microbiome communities using PCA and PERMANOVA. As expected, samples collected from different niches – dental plaque, saliva, or dorsum of the tongue – clustered apart and differed significantly in their profiles (Figures 8A,B). Saliva samples showed significantly higher bacterial diversity than the other two niches (Figures 8C,D). When all samples per niche and study group were assessed by the study visit, the analysis showed that microbiome composition significantly changed in time across all niches (Figure 9). Irrespective of the sample type, samples collected during the experimental gingivitis period (V4 and V5) differed most from the samples collected before or after this period. Additionally, significant changes between the baseline (visit 1) and visit 2 after the first week of the wash-in period with the test product use were observed in microbial profiles of saliva from group A and tongue from groups A and C.

The most pronounced changes in time were nonetheless observed in plaque samples, where in all groups nearly the same anaerobic taxa increased in their proportion during the experimental gingivitis period (Figure 9). Additional to the change between V3 and V4 (first week of the experimental gingivitis period), in groups B and C there was a further significant change between week 1 and week 2 of experimental gingivitis (V4 and V5), while no change in microbiome was observed in the samples from group A.

In saliva, the changes in time were less pronounced, though still significant (A: *p* = 0.0001; B: *p* = 0.0001; C: *p* = 0.0001) (Figure 9). Again, the differences were due to the experimental gingivitis phase. However, in saliva, unlike plaque, some differences in the taxa that changed in their proportion during the experimental gingivitis period were observed among the groups. In group A this was associated with principal component PC3 and with a higher proportion of four zOTUs classified as genus *Leptotrichia* (#26, #94, #117, #55), *Lactobacillus* zOTU #143, and *Abiotrophia defectiva* zOTU#42 (Figure 9). In group B, changes during experimental gingivitis were associated with PC3 and a higher proportion of *Leptotrichia* (zOTU #73, #26), *Fusobacterium* zOTU #43, *Capnocytophaga sputigena* zOTU #62, and *Aggregatibacter* sp. oral taxon 458/512 zOTU #40. In saliva samples from group C, experimental gingivitis was associated with a higher proportion of *Aggregatibacter* sp. oral taxon 458/512 zOTU #40, *Prevotella nigrescens* zOTU #116, *Fusobacterium* zOTU #43, #107, *Selenomonas* zOTU #115, and *Leptotrichia* zOTU #55. In other words, these findings indicate that although direct inter-group comparisons did not show any differences among the three groups, assessments in time within each group indicate that slightly different microbial shifts occurred during the experimental gingivitis period in different groups of saliva samples. Besides the increase in genus *Leptotrichia* (anaerobic Gram-negative rod from class Fusobacteria, associated with mature dental plaque) in saliva from all groups, the samples from group A showed an increase in the proportion of *Lactobacillus* (a likely constituent of one of the test products) and *Abiotrophia defectiva* (facultative anaerobic Gram-positive coccus, nutritionally-dependent on other taxa), while in groups B and C increases in anaerobic and capnophilic taxa, all associated with mature dental plaque, were observed.

The tongue samples showed the least though still statistically significant changes induced by experimental gingivitis. Unlike for saliva and plaque samples, the PCA plots on tongue samples did not reveal any obvious sample clustering by the principal components (Figure 9). Only in group B the tongue sample composition was significantly different between baseline and visit 4 (1 week of experimental gingivitis), while all three groups differed from the baseline after the second week of the gingivitis phase (visit 5).

**Figure 10:**
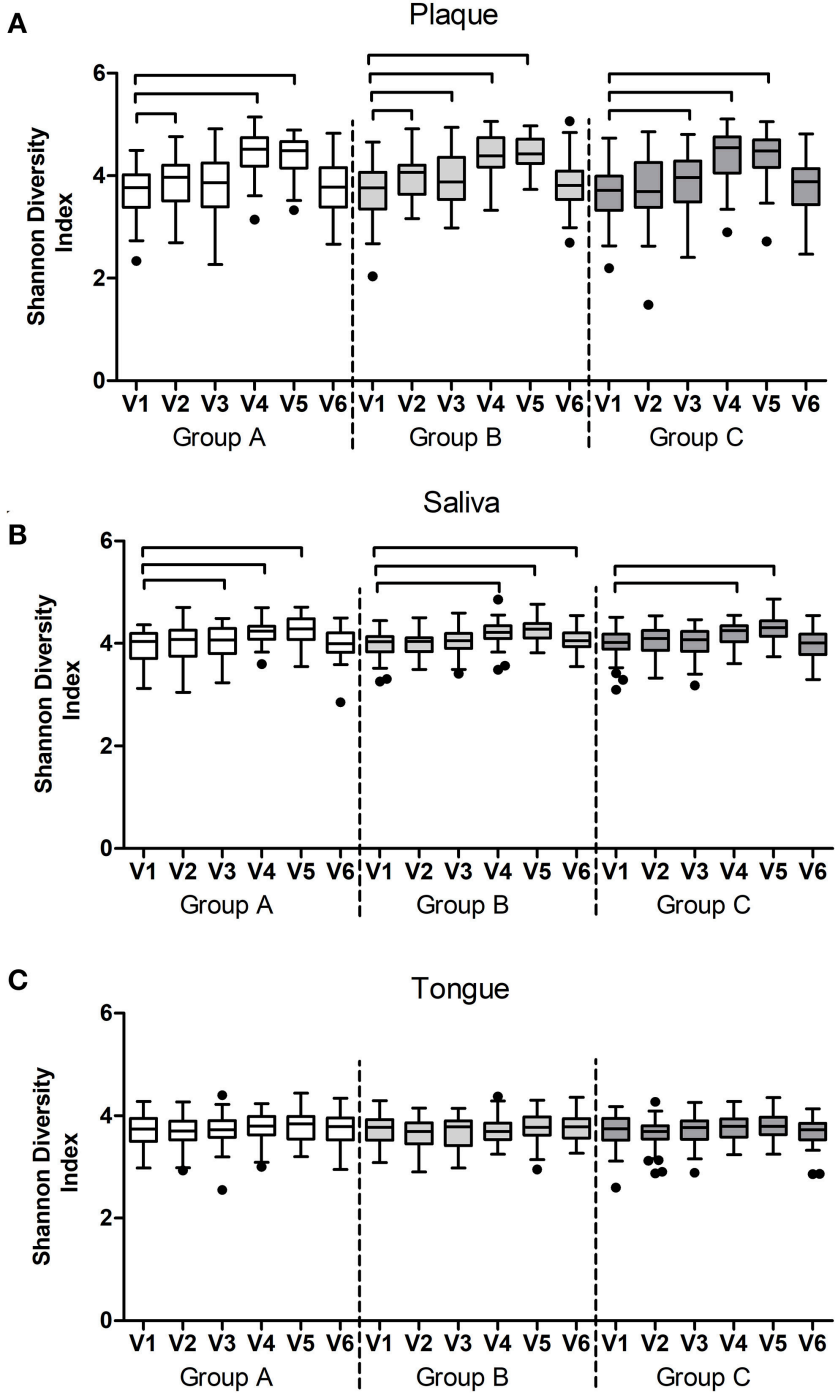
Shannon Diversity Index per study group and visit of **(A)** plaque, **(B)** salivary, and **(C)** tongue microbiome samples. The connectors connect the visits that differed significantly (*P* < 0.05, Wilcoxon Signed Ranks test) from the baseline visit.

Within each group, microbial diversity (species richness and Shannon Diversity Index) of plaque and saliva changed significantly in time, while the diversity of the tongue microbiome remained unchanged (Figure 10). In plaque and saliva, diversity increased significantly in all three groups during the experimental gingivitis phase (V4 and V5). In plaque, diversity increased also during the normal oral hygiene period, from V1 to V2 (groups A and B) and from V1 to V3 (groups B and C) when subjects were exposed to the lozenges (Figure 10).

## Discussion

In this randomized triple-blind and placebo-controlled clinical trial, we assessed the effects of two bacterial strains (formerly known as lactobacilli) that were selected from previous *in vitro* tests as potential probiotics for the oral ecosystem using the experimental gingivitis study design. The primary study outcome, changes in gingival bleeding after the 2 weeks of abstaining from toothbrushing, was not affected by the exposure to the test lozenges in comparison to the placebo group. However, gingival health in individuals from the groups exposed to the test products recovered better from experimental gingivitis than the individuals in the placebo group.

Besides the primary and secondary outcomes, we performed ancillary analyses within each group, in time. Here we found that the two test products inhibited pro-inflammatory cytokine IL-1ß production, measured in saliva, during the experimental gingivitis period. It has been shown that the IL-1ß level increases in saliva during experimental gingivitis and correlates with plaque and gingival indices [27]. Our findings indicate that the tested strains have an anti-inflammatory potential not only *in vitro* [12] but also *in vivo*. The faster recovery in gingival health after the experimental gingivitis in the test groups compared to placebo also strengthens this beneficial potential. Another pro-inflammatory cytokine – PGE2 – did not show measurable changes in saliva during the experimental gingivitis in any of the groups. The latter finding might be explained due to the low concentration measured in saliva and the higher association of PGE2 with periodontitis than gingivitis [26].

Exposure to *L. paracasei* (group A) during the period of normal oral hygiene (the wash-in phase of the study) led to a lower plaque pH, a lower proportion of mature, red fluorescing plaque in comparison to the baseline samples, and changes in microbial profiles of saliva and tongue. Red fluorescence of dental plaque increases with the increase in plaque mass and age [18] and is associated with a mature, anaerobe-rich microbial composition [19, 43]. Our findings suggest that exposure to *L. paracasei*-containing lozenges modulated the ecological properties of the oral ecosystem.

Group A (*L. paracasei)* differed from the other two groups also in the response to experimental gingivitis: while in groups B (control group) and C (*L. plantarum)* microbial composition of dental plaque changed significantly between the first and the second week of experimental gingivitis, the microbial profiles in group A, exposed to *L. paracasei*-lozenges, remained stable. Additionally, no changes in bacterial DNA concentration were observed in group A throughout the entire study, while bacterial DNA increased significantly in the other two groups during abstaining from oral hygiene in comparison to the baseline. Although no differences among the groups in the changes in plaque amount were measured, our findings suggest that the *L. paracasei* test strain has a potential for plaque modulating capacity and should be investigated further. In group A plaque samples, fungal DNA load (fungal DNA relative to bacterial DNA concentration) increased slightly though statistically significantly during the wash-in period of the study. Since we did not assess fungal community composition, the biological relevance of this finding is unclear and requires further study. It has been suggested that fungi play a significant role in maintaining resilient oral microbiota [44].

The microbial composition of the tongue is known to be the most stable among the oral niches and overall in the human body [45]. Nevertheless, the microbial composition of the tongue coating changed significantly during our study where subjects were abstaining from oral hygiene for 2 weeks. However, after the first week of non-brushing, only in the placebo group and not in the test groups the microbial profiles differ from the baseline. This indicates that the test products may have enhanced the resilience of the tongue microbiome.

An unexpected and very interesting finding was the notable reduction of bacterial DNA in the tongue samples exposed to the test lozenges (group A and C) throughout the entire study period, in comparison to the baseline. This could not be attributed to the mechanical removal by the lozenge, since similar effects were not observed in the placebo group. A potential explanation could be that exposure to the test strains has resulted in bacterial loosening and detachment from the tongue coating. The exact mechanisms and potential of the test strains against halitosis [46], not assessed here, require further study.

Limited adherence and retention time of the probiotics in the oral cavity is a known concern [47]. The concentration of both test strains increased significantly during the visits when the subjects were exposed to the test products in the saliva, plaque, and tongue samples of our study. Among the three types of samples, saliva showed the largest counts of the test strains, and 2 weeks after the study still contained a higher concentration of the *L. plantarum*-strain in the group C samples than in the other two groups, indicating that low numbers of this test strain were able to survive in the oral cavity at least 2 weeks beyond the period of direct exposure.

The present study was performed with the well-known experimental gingivitis protocol [4]. However, such a clinical study also has its limitations, which means that extrapolation of the results should be done with caution [48]. The most important aspects are that the population of this study was young and healthy, which makes it difficult to estimate the magnitude of the effects of this intervention in a less healthy or older population. An attempt has been made to limit this effect by applying the experimental gingivitis model, but the effects of experimental gingivitis have not been studied in e.g., patients with a high caries risk [2]. Another point of attention is the frequency of exposure to the lozenges. Here the study participants took three lozenges per day, while this could be difficult to achieve during daily life. For more convenient use, another method of administration of the probiotics could be considered. In addition, the exposure to the lozenges was limited to 4 weeks. Regular use of the lozenges for longer periods might lead to more pronounced effects.

Some observations, unrelated to the test products, were made in this study regarding the study design. First, we observed a significant increase in plaque index in the placebo and one of the test groups (group C, *L. plantarum*) during the wash-in phase, when subjects were asked to continue with their habitual oral hygiene measures. This could be due to a Hawthorne effect [49], where subjects might have temporarily increased their oral hygiene habits before the start of the study, to be able to pass the screening, and lowered to their habitual level once participating in the study. Another observation, relevant for the experimental gingivitis study design, was that none of the clinical indices – PI, MGI, and BOMP – recovered entirely to the baseline levels after the 2 weeks of the recovery phase. Although 2 weeks of recovery is a commonly applied protocol [50], participants of studies with experimental gingivitis design should be followed for a longer period of time.

This study is one of the few large RCTs investigating the effects of probiotics on both clinical and oral microbiome characteristics before, during, and after an experimental gingivitis intervention. The relatively short duration and the specific study population prohibit conclusive statements. However, the two tested lozenges with the specific *L. paracasei* or *L. plantarum* strains did show potential for beneficial effects for the oral health of the host during experimental gingivitis stress to the oral ecosystem and require further studies on their effects in modulating the oral ecology.

## Data Availability Statement

The sequencing data is available in the NCBI SRA Database, BioProject: PRJNA796964. The names of the repository/repositories and accession number(s) can be found below: NCBI; PRJNA796964.

## Ethics Statement

The studies involving human participants were reviewed and approved by the Ethics Committee of Slotervaartziekenhuis & Reade (METC nr. P1815; Dutch CCMO research protocol nr. NL65326.048.18; registration in the Netherlands Trial Register NL6951). The participants provided their written informed consent to participate in this study.

## Author Contributions

BB, MB, BF, TR, and MG contributed to data acquisition and drafted and critically revised the manuscript. CV, SW, NR, TS, WC, and EZ contributed to conception, design, data acquisition, and drafted and critically revised the manuscript. All authors gave final approval and agreed to be accountable for all aspects of the work.

## Funding

These authors had no involvement in the final evaluation of the data or the discussion of the article. This study received funding from Johnson and Johnson Consumer Inc. (USA) grant number 593495, 2016. This funder was not involved in the study design, collection, analysis, interpretation of data, the writing of this article, or the decision to submit it for publication.

## Conflict of Interest

TR and BF are employed by VivaCell Biotechnology GmbH. TS is employed by Symrise AG. MG is a former employee of Symrise AG and is currently employed by Spindiag GmbH. The remaining authors declare that the research was conducted in the absence of any commercial or financial relationships that could be construed as a potential conflict of interest. This study received funding from Symrise. The funder (represented by TS and MG) had the following involvement with the study: interpretation of findings and writing of the article. These authors had no involvement in the final evaluation of the data or the discussion of the article.

## Publisher's Note

All claims expressed in this article are solely those of the authors and do not necessarily represent those of their affiliated organizations, or those of the publisher, the editors and the reviewers. Any product that may be evaluated in this article, or claim that may be made by its manufacturer, is not guaranteed or endorsed by the publisher.
