# Supplementary material for: The Evaluation of the Effects of Two Probiotic Strains on the Oral Ecosystem: A Randomized Clinical Trial
Source: Front Oral Health. 2022 Mar 30;3:825017. doi: 10.3389/froh.2022.825017 (PMC9007728; doi:10.3389/froh.2022.825017)
Supplement: Supplementary file 1 [file Data_Sheet_1.docx]

Supplementary Material

# Supplementary Figures and Tables

## Supplementary Figures


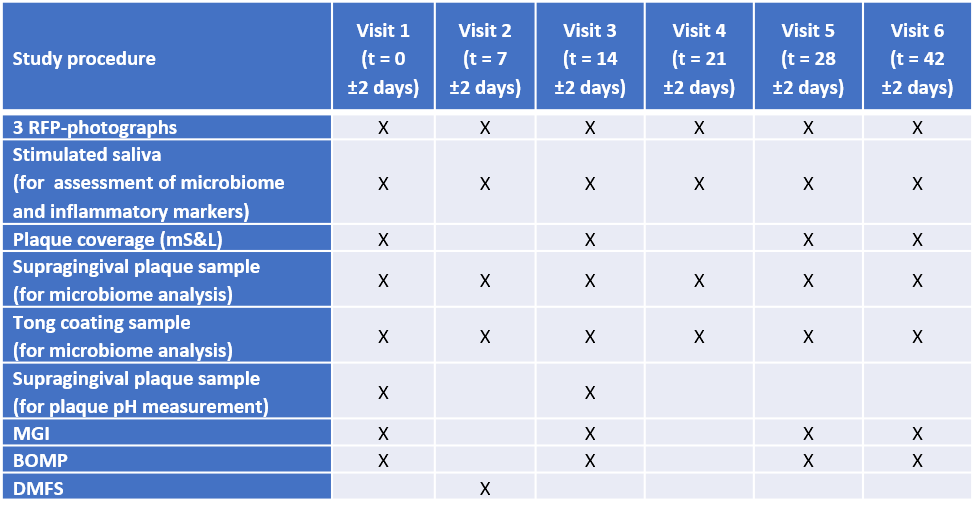


**Supplementary figure 1.** Study procedures per individual visit. RFP – red fluorescing plaque; mS&L – modified Silness & Löe plaque index; MGI – modified gingival index; BOMP – bleeding on marginal probing; DMFS – decayed, missing, filled surfaces.


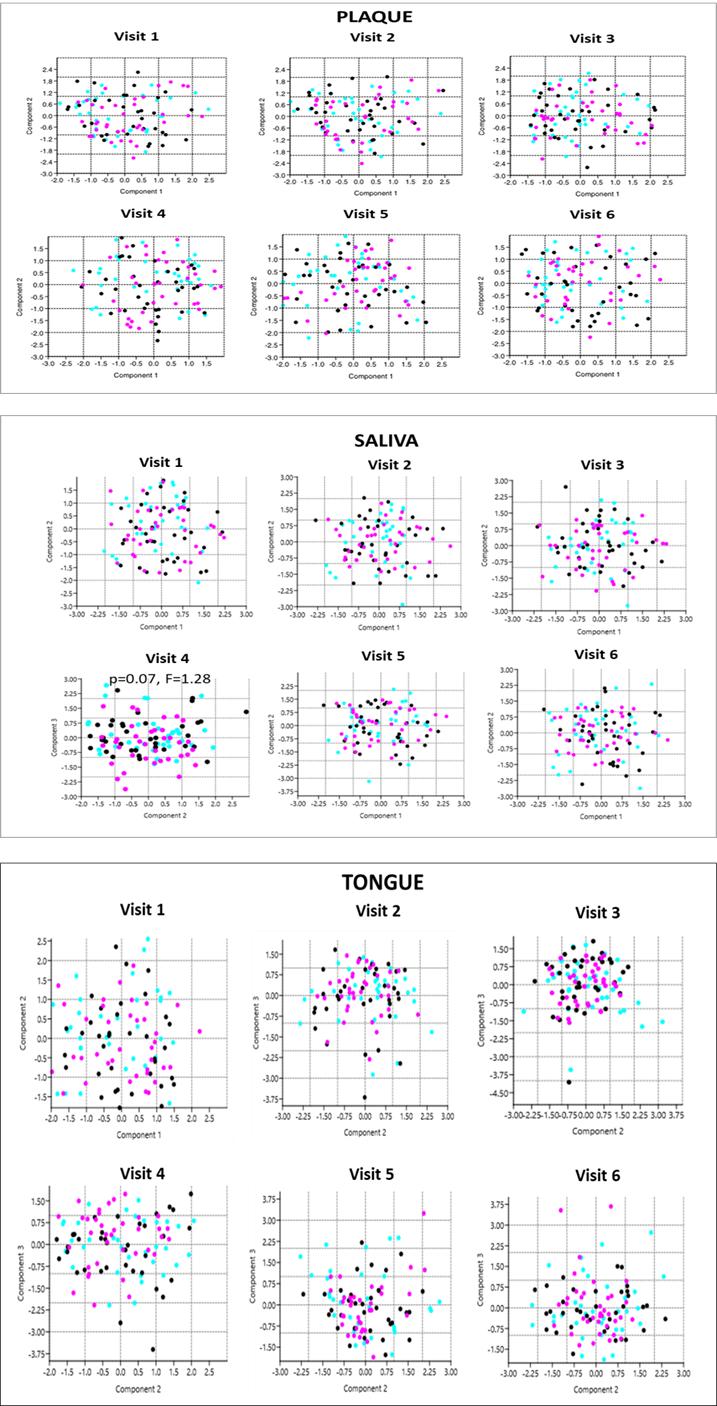
**Supplementary figure 2.** PCA of microbiome profiles per niche and per visit. No differences in microbial profiles (p>0.05, PERMANOVA) among the groups per individual visit and niche. In saliva samples collected at V4, nearly significant (p=0.07, F=1.28) change was observed. Aqua – Group A, Black – group B, Fuchsia – Group C.


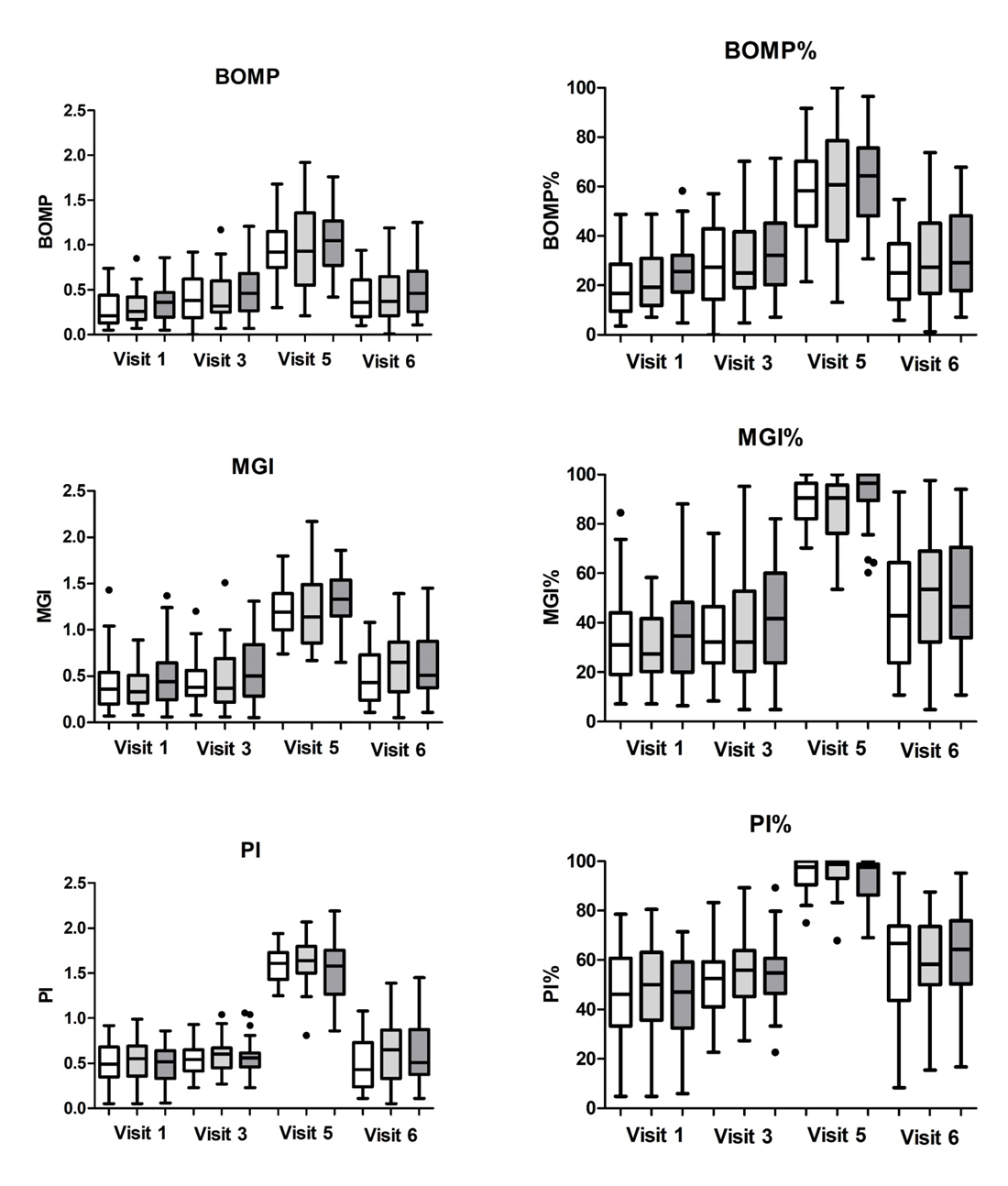


**Supplementary Figure 3.** Gingival bleeding (BOMP, %BOMP), gingival inflammation (MGI, %MGI), plaque index (PI, %PI) per visit and study group. White boxes – group A, light grey boxes – group B, dark grey boxes – group C.


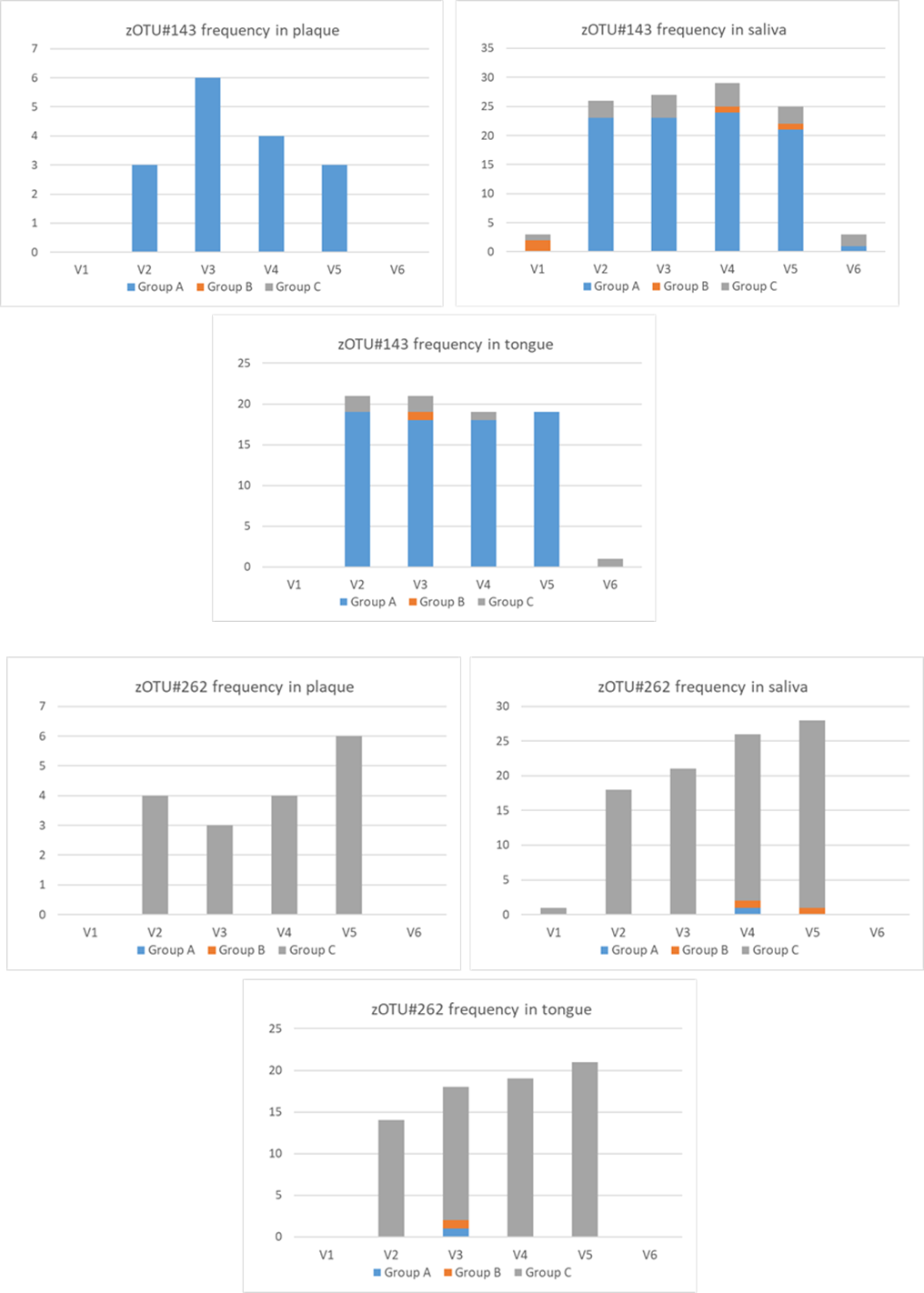


**Supplementary Figure 4.** Frequency (nr of samples with at least 1 read) of two major zOTUs – zOTU#143 and zOTU#262 - in the sequencing dataset that were assigned to genus *Lactobacillus* per visit and study group by niche.

## Supplementary Tables

**Supplementary Table 1.** Nucleotide sequence of primer and probes specific for *L. plantarum* and *L. paracasei* (purchased from Microsynth AG, Balgach, Switzerland).

| Target strain | Target gene | Primer or probe | Sequence (5’ -> 3’) | Melting T (°C) | Amplicon length (bp) | Final concentration (nM) |
| --- | --- | --- | --- | --- | --- | --- |
| *L. plantarum* | 23S rDNA | Lplan^1^-F^2^ | 5’-TGTGCCTACAATAAGTCAGAGC-3’ | 63.0 | 157 | 800 |
|  |  | Lplan-P^3^ | **5’-FAM-**ATGCGTGATGGCGTGCCTTTTGTAGA –**BHQ1-3’** | 69.9 | 157 | 200 |
|  |  | Lplan-R^4^ | 5’-GGTCTATAACCTCGTACTCAAAACG-3’ | 63.3 | 157 | 800 |
| *L. paracasei* | 16S rDNA | Lpara^5^-F | 5’-GCACCGAGATTCAACATGG -3’ | 61.8 | 117 | 700 |
|  |  | Lpara-P | **5’-FAM-**ACACGTGGGTAACCTGCCCTTAAGTGG-**BHQ1-3’** | 70.6 | 117 | 200 |
|  |  | Lpara-R | 5’-GGTTCTTGGATC(Y)TATGCGGTATTAG-3’ | 63.4 | 117 | 700 |

^1^*Lactobacillus plantarum*, ^2^Forward Primer, ^3^Probe, ^4^Reverse Primer, ^5^*Lactobacillus paracasei*

**Supplementary Table 2.** Reasons for exclusion by phone / e-mail.

| Reason for exclusion | Frequency |
| --- | --- |
| Did not attend the physical screening (without reason) | 3 |
| Piercing | 1 |
| Ramadan | 2 |
| Used a night guard | 1 |
| Recent use of antibiotics | 4 |
| Breastfeeding | 1 |
| Working / studying at ACTA | 1 |
| Diabetes | 1 |
| Was not allowed to use probiotics | 1 |
| Lactose intolerance | 3 |
| Had elaborate dental treatments planned | 1 |
| It turned out the travel time was too long | 1 |
| Was not able to attend all visits | 6 |
| Was not able to be present during the day at ACTA | 2 |
| Not interested in the study anymore | 2 |
| Did not want to stop oral hygiene | 2 |
| Used medication | 10 |
| Did not attend the dentist for more than a year | 1 |
| No reason given (people are free to renounce participation) | 11 |

**Supplementary Table 3.** Summary of the reasons for exclusion during the screening visit.

| Reason for exclusion | Frequency |
| --- | --- |
| Periodontal attachment loss | 2 |
| Active caries lesion | 7 |
| Alveolitis | 1 |
| Use of anti-inflammatory medication | 2 |
| Could not attend all research visits | 2 |
| Reserve | 2 |

**Supplementary Table 4.** Summary of minor protocol deviations.

|  | Group A | Group B | Group C |
| --- | --- | --- | --- |
| Nr of minor deviations | 21 | 36 | 32 |
| Nr of subjects involved | 17 | 23 | 24 |
| Impact for any secondary output parameter(s) | 1 | 1 | 5 |

**Supplementary Table 5.** Summary of adverse events in ITT/SA population (N=111).

|  | Adverse Events | Group A | Group B | Group C |
| --- | --- | --- | --- | --- |
| Total nr of events (subjects) | | 13 (10) | 15 (10) | 13 (11) |
| Nr of events (subjects) possibly related with treatment: | | 5 (4) | 7 (4) | 6 (5) |
|  | Events (subjects) with aphtha (MEDDRA code 10067589) | 5 (4) | 1 (1) | 5 (4) |
|  | Events (subjects) with sensitive teeth (MEDDRA code 10079311) | 0 | 6 (3) | 0 |
|  | Events (subjects) with stomach cramps (MEDDRA code 10049901) | 0 | 0 | 1 (1) |

**Supplementary Table 6.** Product tolerance scores per visit and group (N=111, ITT/SA population). The subjects rated their experience with lozenges between 0 (very unpleasant) and 10 (very pleasant).

| Study visit | Group A | Group B | Group C |
| --- | --- | --- | --- |
| Visit 2  Mean (SD)  Median (range) | 6.8 (0.9)  7 (5-9) | 7.1 (1.3)  7 (3-10) | 6.7 (1.3)  7 (4-10) |
| Visit 3  Mean (SD)  Median (range) | 6.7 (1)  7 (4-9) | 7.1 (1.3)  7 (3-10) | 6.8 (1.2)  7 (4-10) |
| Visit 4  Mean (SD)  Median (range) | 6.9 (0.7)  7 (5-8) | 7.1 (1.2)  7 (5-9) | 7 (1.2)  7 (4-10) |
| Visit 5  Mean (SD)  Median (range) | 6.9 (0.8)  7 (5-9) | 7 (1.2)  7 (5-10) | 7 (1.1)  7 (5-10) |

**Supplementary Table 7.** zOTUs that were taxonomically assigned as *Lactobacillus*, and the total number of reads of each zOTU in the subsampled dataset.

| zOTU_ID | Nr of reads |
| --- | --- |
| *Zotu143;g__Lactobacillus* | 7820 |
| *Zotu262;g__Lactobacillus;(s__pentosus/s__plantarum)* | 1780 |
| *Zotu1419;g__Lactobacillus;(s__pentosus/s__plantarum)* | 233 |
| *Zotu455;g__Lactobacillus;s__ultunensis* | 230 |
| *Zotu535g__Lactobacillus;s__gasseri* | 197 |
| *Zotu571;g__Lactobacillus;(s__pentosus/s__plantarum)* | 190 |
| *Zotu539;g__Lactobacillus* | 190 |
| *Zotu518;g__Lactobacillus;s__salivarius* | 187 |
| *Zotu522;g__Lactobacillus* | 127 |
| *Zotu670;g__Lactobacillus;s__fermentum* | 72 |
| *Zotu721;g__Lactobacillus;(s__oris/s__panis/s__reuteri_genosp._1/s__reuteri_genosp._2)* | 48 |
| *Zotu694;g__Lactobacillus* | 44 |
| *Zotu894;g__Lactobacillus* | 42 |
| *Zotu751;g__Lactobacillus* | 34 |
| *Zotu737;g__Lactobacillus* | 29 |
| *Zotu1039;g__Lactobacillus;s__ultunensis* | 22 |
| *Zotu1142;g__Lactobacillus* | 21 |
| *Zotu1064;g__Lactobacillus;(s__buchneri/s__kisonensis/s__rapi)* | 19 |
| *Zotu1132;g__Lactobacillus;s__iners* | 14 |
| *Zotu2170;g__Lactobacillus;(s__oris/s__panis/s__reuteri_genosp._1/s__reuteri_genosp._2)* | 10 |
| *Zotu1505;g__Lactobacillus* | 9 |
| *Zotu1935;g__Lactobacillus* | 9 |
| *Zotu1846;g__Lactobacillus;(s__buchneri/s__kisonensis/s__rapi)* | 8 |
| *Zotu1229;g__Lactobacillus;s__fermentum* | 7 |
| *Zotu1315g__Lactobacillus* | 7 |
| *Zotu1382;g__Lactobacillus;(s__oris/s__panis/s__reuteri_genosp._1/s__reuteri_genosp._2)* | 6 |
| *Zotu1235;g__Lactobacillus;s__ultunensis* | 5 |
